# Supplementary material for: Reporting and methodological quality of systematic reviews and meta-analysis with protocols in Diabetes Mellitus Type II: A systematic review
Source: PLoS One. 2020 Dec 16;15(12):e0243091. doi: 10.1371/journal.pone.0243091 (PMC7743973; doi:10.1371/journal.pone.0243091)
Supplement: S1 File — (PDF) [file pone.0243091.s001.pdf]

The Reporting and Methodological Quality of Systematic Reviews and Meta-Analysis with Protocols in Diabetes Mellitus Type II: A Review of Systematic Reviews (Protocol)

Lead PI: Dr Daniel Rainkie

Co-Authors: Ms Zeinab Abedini, Ms Nada Abdelkader

Table of Contents

Methods ... 5
Eligibility Criteria ... 5
Search Strategy and Identification of Reviews ... 5
Selection of Reviews ... 5
Search Strategy and Identification of Protocols ... 5
Data Items ... 5
Data Collection Process ... 8
Outcomes ... 8
Primary outcomes ... 8
Secondary outcomes ... 8
Data Synthesis ... 9
Protocol Registration ... 9
Timeline ... 9
Funding sources/sponsors ... 9
Conflicts of interest ... 9
Language ... 9
Stage of the Review (status) ... 9
Date of publication of this version ... 10
Acknowledgements ... 10

Introduction

Evidence-based medicine is the integration of the best available research evidence with clinician's clinical experience and patient health outcomes.[1] Clinician's base their clinical decisions and policies on reliable research in order to provide informed healthcare decisions. Thus, ensuring the best care provided, and standardizing healthcare quality. A considerable number of health-related trials and studies is being published on a daily basis. Therefore, in an attempt to stay up-to-date with the continuous rising number of publications; one must read an insuperable number of articles daily which is not practical and time consuming. As a result, systematic reviews were developed to summarize the available evidence for clinicians.[2] As per Cochrane Collaboration "A systematic review attempts to identify, appraise and synthesize all the empirical evidence that meets pre-specified eligibility criteria to answer a specific research question". While they defined meta-analysis as the combination of individual studies results to produce an overall statistic; which aims at providing a more precise estimate of the effects of an intervention reducing uncertainty.[3] Systematic reviews and meta-analyses are essential for updating healthcare providers, and for summarizing all the relevant research pertaining to a specific question. Moreover, they are considered the starting point of clinical practice guidelines; through recognizing the gaps which could attract funders to support new research areas. Additionally, they provide comprehensive evidence for policymakers which enables them to determine contributing factors, risks, benefits, and harms of diverse therapy regimens.[4] Systematic reviews and meta-analysis are considered the highest quality of evidence available in the hierarchy of the evidence-based medicine pyramid due to its large sample size and its robust methodology that handles bias in the included studies through results pooling.[5] However, when systematic reviews and meta-analysis are poorly reported, the review becomes useless to clinicians as it's quality cannot be assessed. Furthermore, the applicability of its conclusions become difficult and in doubt.[6] Not to mention, systematic reviews and meta-analyses are becoming increasingly common. As per the available data, at least 2,500 new systematic reviews are reported in MEDLINE per year; whereas, 11 new reviews were estimated to be published daily in 2010.[7,8]

These reviews must be comprehensive, transparent, reliable and reproducible could improve the effectiveness of the evidence. Unfortunately, there were inconsistencies recognized in systematic reviews and meta-analyses which lead to the development of several guidelines and checklists to ensure the improvement of both completeness of reporting and the quality of the reviews.[9,10] Completeness of the reporting of the manuscript was assessed through QUOROM (QUality Of Reporting Of Meta-analyses) Statement with detailed reporting recommendations which was published in 1999.[11] In 2009, aiming for Several conceptual and practical advances in the methodology of systematic reviews; the QUOROM was updated and renamed as the Preferred Reporting Items for Systematic reviews and Meta-Analyses (PRISMA) Statement.[12,13] However, all systematic reviews must be conducted based on their pre-specified eligibility criteria and methodological approach as mentioned per protocol. The protocol construction is crucial as it ensures that the systematic review is planned and documented explicitly ensuring complete transparency and clarifying the data extraction process while discussing potential problems that may arise. Thus, promoting the methodology consistency, content integrity, transparency, reproducibility and reliability of the systematic review. Additionally, deviations and sources of bias can be identified by the readers allowing them to assess the reliability of the results and their applicability. Possible sources of bias could be relating to the methodology, results or conclusions where selective reporting bias is one of the most recognized by clinicians' and can affect their decisions.[14,15,16] As a result, PRISMA for Protocol (PRISMA-P) was developed as an extension of the

PRISMA statement as a guideline and a 17-item checklist in 2015.[17,18] Nevertheless, the Completeness of reporting cannot assure the quality of the data reported. Hence, quality improvement tools were developed to enable the assessment of systematic review's quality using a standardized method. In 2007, the A MeaSurement Tool to Assess systematic Reviews (AMSTAR) was published; which is one of the most commonly used tools.[19,20] Later on, the AMSTAR-2 tool was published in 2017; as it could be used for both systematic reviews of randomized or non-randomized trials along with a more comprehensive user guide with an overall rating based on weaknesses in critical domains. The revised AMSTAR 2 tool has 10 of the original domains and it's composed of 16-items in total. On the other hand, the AMSTAR2 has simpler response categories when compared to the original AMSTAR tool, but it's not intended to generate an overall score.[21] Despite of the available guidelines and quality assessment tools and checklists; the reporting and methodological quality of systematic reviews remain inconsistent. In 2013, a review was conducted under the following title "Association of Study Quality with Completeness of Reporting: Have Completeness of Reporting and Quality of Systematic Reviews and Meta-analyses in Major Radiology Journals Changed Since Publication of the PRISMA Statement?". It concluded that in major radiology journal studies; a significant improvement in complete reporting of systematic reviews and meta-analyses which was assessed by PRISMA proving a strong association between adequate reporting and the study's quality which was assessed using AMSTAR tool.[22] However, despite the large number of studies conducted to identify the association between complete reporting and methodological quality of systematic reviews; none was conducted using the AMSTAR2 as it was recently published. Nevertheless, none of the past studies linked the reporting of protocols and reporting of manuscripts with methodological quality using the three checklists (PRISMA, PRISMA-p, AMSTAR2). Thus, this gap must be explored and addressed which is the rationale of our review. Since, it became evident that more research in this area must be done to identify if there's a correlation between adequate reporting of protocols and adequate reporting of the manuscript itself and whether that will reflect on the quality of the study revealing a positive correlation between the tools. Identifying such relations between the tools will enable clinicians to understand better and assess the available literate properly prior to integrating it in practice and shaping their decisions and guidelines based on it. Last but not least, diabetes mellitus type two is identified as one of the most prevalent diseases in Qatar and the world. It's the leading cause of many disabilities, complications and premature deaths.[23,24] Hence, lots of systematic reviews and meta-analyses were published regarding diabetes due to its prevalence which is influencing clinical practice and clinical decisions. We hypothesized that there is an association between adequate reporting in protocols and adequate reporting in manuscripts and that there is a positive correlation between the reporting and transparency of reviews and the quality of systematic reviews and meta-analysis. Our primary objective is to identify the correlation between PRISMA-p and PRISMA checklists' scores and our secondary objectives are to identify the association between PRISMA and AMSTAR2 and finally between PRISMA-p and AMSTAR2.

Methods

Eligibility Criteria

In this review of systematic reviews and meta-analysis, we will include only systematic reviews and/or meta-analysis with available protocols that assess or compare between any pharmacological hypoglycemic agents for the management of diabetes mellitus type II.

Articles will be excluded if SR/SRMAs without an available protocol, they were Cochrane reviews, select article meta-analyses (e.g. a meta-analysis of phase III trials of new antidiabetic agents in which a systematic review was not evident) or guidelines. Cochrane reviews were excluded because as a peer-reviewed protocol is mandatory to publish in Cochrane.

Search Strategy and Identification of Reviews

We will search PubMed and EMBASE for any systematic reviews or meta-analyses of hypoglycemic agents for the management of T2DM for any outcome. We will limit the date of publication starting from 1st of January 2015 up till present as the PRISMA-P checklist was published in 2015. Our search will be limited to English publications only as we have no means of accurate translation.

MeSH terms that will be used to search PubMed are ("Diabetes Mellitus, Type 2"[Mesh] AND "Hypoglycemic Agents"[Mesh]) with search limits including systematic reviews and meta-analysis only, English only and since Jan 1st, 2015. The search terms which will be used for the EMBASE search are as follows: (AND ('meta analysis'/de OR 'systematic review'/de) AND 'non insulin dependent diabetes mellitus'/de AND 'antidiabetic agent'/de AND (2015:py OR 2016:py OR 2017:py OR 2018:py OR 2019:py)) including the same limits used for PubMed search.

Selection of Reviews

All authors will independently complete the search and screen by title and abstract for relevant inclusion and exclusion criteria. Results will be collated in to a Microsoft Excel database. Duplicates will be removed and protocols for the identified SR/SRMAs will be sought for. We expect that there will be a large number of included articles based on our cursory search and an estimate of 50% protocol availability. Therefore, we will use a computer-generated randomization tool in Microsoft Excel to randomize all identified articles and then start at the top of the list and search for the article's protocol. We aim to include 100 articles, a sample of convenience, for analysis.

Search Strategy and Identification of Protocols

Two reviews will independently identify relevant protocols based on searching the manuscript, manuscript bibliography, supplementary material, searching PROSPERO and a Google search. If a manuscript stated that a protocol was available by contacting the authors, we will gather this information but will not contact the authors. The rationale for this is that the target audience using these SR/SRMAs to make treatment decisions will likely not contact an author to review the protocol before using these results in practice.

Data Items

The following data items will be collected for each SR/SRMA identified:

1. Title
2. Study authors
3. Reference
4. PubMed / EMBASE URL
5. Publication year
6. Inclusion criteria: SR or SRMA, includes hypoglycemic agents, T2DM population
7. Protocol availability: Journal website/supplementary material, bibliography, PROSPERO, Google search, author contact

For each protocol, the PRISMA protocol (PRISMA-P) checklist will be applied with the following items:

1a. Title Identification
2. Registration
3a. Contact
3b. Contributions
5a. Sources
5b. Sponsor
5c. Role of sponsor or funder
6. Rationale
7. Objectives
8. Eligibility criteria
9. Information sources
10. Search strategy
11a. Data management
11b. Selection process
11c. Data collection process
12. Data items
13. Outcomes and prioritization
14. Risk of bias in individual studies
15a. Data synthesis – criteria for quantitative synthesis
15b. Planned summary measures, handling data, methods of combining data, exploration of consistency
15c. Proposed additional analyses (e.g. sensitivity, subgroup or meta-regression)
15d. If quantitative synthesis is not appropriate, the type of summary planned
16. Meta-biases
17. Confidence in cumulative evidence

In the PRISMA-P checklist we excluded items 1b, updates, and 4, amendments, because many protocols will not have updates or amendments or that it would not be clear and the reviewer would have no way of confirming or rejecting that the protocol being assessed is an update or contains the correct amendments. Therefore, these items are irrelevant for the purposes of this study.

For each SR/SRMA identified, the PRISMA checklist will be applied to determine the completeness of reporting. All 27 items will be assessed in the PRISMA checklist:

1. Title
2. Structured Summary
3. Rationale
4. Objectives
5. Protocol and registration (methods)
6. Eligibility criteria (methods)
7. Information sources (methods)
8. Search (methods)
9. Study selection (methods)
10. Data collection process (methods)
11. Data items (methods)
12. Risk of bias in individual studies (methods)
13. Summary measures (methods)
14. Synthesis of results (methods)
15. Risk of bias across studies (methods)
16. Additional analyses (methods)
17. Study selection (results)
18. Study characteristics (results)
19. Risk of bias within studies (results)
20. Results of individual studies (results)
21. Synthesis of results (results)
22. Risk of bias across studies (results)
23. Additional analysis (results)
24. Summary of evidence
25. Limitations
26. Conclusions
27. Funding

For each SR/SRMA identified, the AMSTAR-2 tool will be used to assess the quality of the manuscript using all sources of data. All 16 items will be assessed in the AMSTAR-2 tool:

1. PICO included in research question and inclusion criteria
2. Protocol with explicit statement of methods prior to the conduction of the review and were the deviations from the protocol justified
3. Selection of the study design for inclusion in the review explained by the authors
4. Comprehensive literature search strategy used
5. Study selection in duplicate by the authors
6. Data extraction in duplicate by the authors
7. List of excluded studies with justification
8. Adequate detailed description of the included studies
9. Satisfactory technique used for assessing the risk of bias in individual studies included (RCTs or NRSI as appropriate)
10. Sources of funding for the studies included in the review reported

11. For meta-analysis; the usage of appropriate methods for statistical combination of the results
12. For meta-analysis; the assessment for potential impact of risk of bias in individual studies on the overall results
13. Accounting for the risk of bias in results interpretation and discussion
14. Satisfactory explanation provided and discussion of heterogeneity if present
15. In case of quantitative synthesis; adequate investigation of publication bias (small study bias) and discussion of its impact on the results
16. Authors reported any potential sources of conflict of interest, including any funding they received for conducting the review
17. Summary assessment of the SR/SRMA quality.

Data Collection Process

In order to ensure that the original intent of the PRISMA-P, PRISMA and AMSTAR-2 checklist/tool was kept, all authors read and studied the explanation and elaboration documents associated for each checklist/tool. Items for the PRISMA-P and PRISMA checklists will be given a "yes" completed or "no" not completed. Each item in the AMSTAR-2 tool will be assigned a "yes", "partial yes" or "no" as described by the requirements for each item. Partial yes answers were considered "yes" for the purposes of the summary assessment based on the judgement of the assessors. For items which are not applicable (i.e meta-analysis questions but the article was only a systematic review, criteria will be marked as "yes" for the summary assessment and to help comparisons) Finally, all major and minor criteria will be used to provide a summary assessment of the SR/SRMA (high, moderate, low or critically low) as described in the published tool.[BMJ 2017]

We performed a standard setting process whereby all authors will independently assess the first 10% of all included studies by assessing and discuss 1-2 articles at a time. Any discrepancies of assessment will be resolved by discussion.

After the standard setting process is complete and agreement is > 80% for each checklist/tool, 2 authors will independently assess their assigned articles. The authors will then meet together and come to consensus on each item. Any discrepancies of assessment will be resolved by discussion and by inclusion of the 3rd author.

Outcomes

Primary Outcomes

- Summary of protocol and manuscript completeness of reporting (PRISMA-P and PRISMA) and quality (AMSTAR-2) of SR/SRMAs
- Correlation of completeness of reporting between PRISMA-P and PRISMA checklist scores

Secondary Outcomes

- Association between PRISMA and AMSTAR2 scores and PRISMA-P and AMSTAR-2 scores.

We have no planned subgroup or sensitivity analyses.

Post-hoc Analysis [update May 2019]

After performing our additional analyses, it was apparent that the rates of overall quality assessments were considered "low" or "critically low" for most articles, primarily due to the poor reporting and

description of the protocol (AMSTAR 2 item 2). On review, we concluded that despite the presence or absence of a protocol and the completeness of the protocol that the results and interpretation of SR/MAs could be deemed appropriate if the other critical criteria of the AMSTAR 2 tool were met. We performed a sensitivity analysis excluding item 2 to determine the change in the summary assessment.

Data Synthesis

Regarding the descriptive statistics; we will tabulate the scores generated by the checklists to generate a mean/median depending on the normality of the data for comparison purposes. The descriptive analysis for normality testing will be done using Kolmogrov-Smirnov statistical test. We will summarize the extracted data from the systematic reviews/meta-analysis included as scores, frequencies, and percentages of reporting for the report characteristics. Inter-rater reliability will be assessed using Cohen's kappa coefficient statistical test and percent agreement. The primary objective will be assessed using Pearson's chi-squared test or Spearman rho correlation test (depending on data distribution and normality); while the secondary objective of association between PRISMA vs AMSTAR-2 and PRISMA-P vs AMSTAR-2 will be assessed using linear regression. A p-value of less than 0.05 will be used as statistical significance. All data will be analyzed using the statistical package SPSS software version 23.0.

Protocol Registration

Our protocol will not be registered with PROSPERO as it does not fit the criteria for registration. This protocol will be published as a supplementary material or available by email.

Timeline

Anticipated or actual start date: Oct 2018

Anticipated completion date**:** Oct 2019 (data collection completed as of May 2019)

Funding sources/sponsors

This project is not funded.

Conflicts of interest

The authors have no conflicts of interests to declare.

Language

English

Stage of the Review (status)

Review-ongoing

Sept 2019 – all outcomes and post-hoc sensitivity analysis completed and reported.

Last update of the protocol is Sept 22, 2019 (update to progress and wording of methods)

Date of publication of this version

Stage	Started	Completed
Preliminary searches	**Yes**	**Yes**
Piloting of the study selection process	**Yes**	**Yes**
Formal screening of search results against eligibility criteria	**Yes**	**Yes**
Data extraction (PRISMA, PRISMA-p)	**Yes**	**Yes**
Methodological quality assessment (AMSTAR2)	**Yes**	**Yes**
Data analysis	**Yes**	**No**

Acknowledgements

Contributions of Authors:

- DR is responsible for conception of the idea, finalizing of the protocol and manuscript.
- DR/ZA/NA are all responsible for drafting the protocol, the data collection, data analysis and drafting of the manuscript.

References

1. Green S. Systematic reviews and meta-analysis. Singapore Med J. 2005;46(6).
2. Ganeshkumar P, Gopalakrishnan S. Systematic reviews and meta-analysis: Understanding the best evidence in primary healthcare. Journal of Family Medicine and Primary Care. 2013;2(1):9.
3. About Cochrane Reviews | Cochrane Library [Internet]. Cochranelibrary.com. 2019 [cited 8 March 2019]. Available from: https://www.cochranelibrary.com/about/about-cochrane-reviews
4. Deville´ WL, Buntinx F, Bouter LM, Montori VM, de Vet HC, et al. (2002) Conducting systematic reviews of diagnostic studies: didactic guidelines. BMC Medical Research Methodology 2: 9.
5. Guyatt G. Users' guides to the medical literature. IX. A method for grading health care recommendations. Evidence-Based Medicine Working Group. JAMA: The Journal of the American Medical Association. 1995;274(22):1800-1804.
6. McGrath T, Alabousi M, Skidmore B, Korevaar D, Bossuyt P, Moher D et al. Recommendations for reporting of systematic reviews and meta-analyses of diagnostic test accuracy: a systematic review. Systematic Reviews. 2017;6(1).
7. Moher D, Tetzlaff J, Tricco AC, Sampson M, Altman DG (2007) Epidemiology and reporting characteristics of systematic reviews. PLoS Med 4: e78. doi:10.1371/journal.pmed.0040078
8. Bastian H, Glasziou P, Chalmers I: Seventy-five trials and eleven systematic reviews a day: how will we ever keep up? PLoS Med 2010, 7(9):e1000326.
9. Ge L, Wang J, Li J, Liang L, An N, Shi X et al. The Assessment of the Quality of Reporting of Systematic Reviews/Meta-Analyses in Diagnostic Tests Published by Authors in China. PLoS ONE. 2014;9(1):e85908.
10. Delaney A, Bagshaw S, Ferland A, Laupland K, Manns B, Doig C. The quality of reports of critical care meta-analyses in the Cochrane Database of Systematic Reviews: An independent appraisal*. Critical Care Medicine. 2007;35(2):589-594.

11. Moher D, Cook DJ, Eastwood S, Olkin I, Rennie D, et al. (1999) Improving the quality of reports of meta-analyses of randomised controlled trials: The QUOROM statement. Quality of Reporting of Meta-analyses. Lancet 354: 1896–1900.
12. Moher D, Liberati A, Tetzlaff J, Altman DG, Group P. Preferred reporting items for systematic reviews and meta-analyses: the PRISMA statement. PLoS Med. 2009;6(7):e1000097.
13. Liberati A. The PRISMA Statement for Reporting Systematic Reviews and Meta-Analyses of Studies That Evaluate Health Care Interventions: Explanation and Elaboration. Annals of Internal Medicine. 2009;151(4):W.
14. Chan AW, Hróbjartsson A, Haahr MT, Gøtzsche PC, Altman DG: Empirical evidence for selective reporting of outcomes in randomized trials: comparison of protocols to published articles. JAMA 2004, 291(20):2457–2465.
15. Kirkham JJ, Altman DG, Williamson PR: Bias due to changes in specified outcomes during the systematic review process. PLoS ONE 2010, 5(3):e9810.
16. Page MJ, McKenzie JE, Kirkham J, Dwan K, Kramer S, Green S, Forbes A: Bias due to selective inclusion and reporting of outcomes and analyses in systematic reviews of randomised trials of healthcare interventions. Cochrane Lib 2014, (10):Art No.:MR000035. doi:10.1002/14651858.MR000035. pub2.
17. Moher D, Shamseer L, Clarke M, Ghersi D, Liberati A, Petticrew M et al. Preferred reporting items for systematic review and meta-analysis protocols (PRISMA-P) 2015 statement. Systematic Reviews. 2015;4(1).
18. Moher D, Shamseer L, Clarke M, Ghersi D, Liberatiˆ A, Petticrew M et al. Preferred reporting items for systematic review and meta-analysis protocols (PRISMA-P) 2015: elaboration and explanation. BMJ. 2016;:i4086.
19. Shea BJ, Hamel C, Wells GA, et al. AMSTAR is a reliable and valid measurement tool to assess the methodological quality of systematic reviews. *J Clin Epidemiol* 2009;62:1013-20. doi:10.1016/j. jclinepi.2008.10.009
20. Shea BJ, Bouter LM, Peterson J, et al. External validation of a measurement tool to assess systematic reviews (AMSTAR). *PLoS One* 2007;2:e1350. doi:10.1371/journal.pone.0001350
21. Shea B, Reeves B, Wells G, Thuku M, Hamel C, Moran J et al. AMSTAR 2: a critical appraisal tool for systematic reviews that include randomised or non-randomised studies of healthcare interventions, or both. BMJ. 2017;:j4008.
22. Tunis A, McInnes M, Hanna R, Esmail K. Association of Study Quality with Completeness of Reporting: Have Completeness of Reporting and Quality of Systematic Reviews and Meta-Analyses in Major Radiology Journals Changed Since Publication of the PRISMA Statement?. Radiology. 2013;269(2):413-426.
23. Al-Kaabi S, Atherton A. Impact of noncommunicable diseases in the State of Qatar. ClinicoEconomics and Outcomes Research. 2015;:377.
24. Qatar [Internet]. Institute for Health Metrics and Evaluation. 2019 [cited 9 March 2019]. Available from: http://www.healthdata.org/qatar
