# Supplementary material for: Reporting and methodological quality of systematic reviews and meta-analysis with protocols in Diabetes Mellitus Type II: A systematic review
Source: PLoS One. 2020 Dec 16;15(12):e0243091. doi: 10.1371/journal.pone.0243091 (PMC7743973; doi:10.1371/journal.pone.0243091)
Supplement: S2 File — (DOCX) [file pone.0243091.s002.docx]

# Excluded Studies Based on Inclusion Criteria

| Title | Study Authors | Reference | Systematic Review or MA only | Hypo-glycemic agent | T2DM |
| --- | --- | --- | --- | --- | --- |
| A meta-analysis of rate ratios for nocturnal confirmed hypoglycaemia with insulin degludec vs. insulin glargine using different definitions for hypoglycaemia. | Heller S, Mathieu C, Kapur R, Wolden ML, Zinman B. | Diabet Med. 2016 Apr;33(4):478-87. doi: 10.1111/dme.13002. Epub 2015 Dec 13. | Select MA |  |  |
| A novel, long-acting glucagon-like peptide receptor-agonist: Dulaglutide | Gurung T., Shyangdan D.S., O'Hare J.P., Waugh N. | Diabetes, Metabolic Syndrome and Obesity: Targets and Therapy (2015) 8 (363-386). Date of Publication: 10 Aug 2015 | Select MA |  |  |
| Achievement of treatment goals with canagliflozin in patients with type 2 diabetes mellitus: a pooled analysis of randomized controlled trials. | Blonde L, Woo V, Mathieu C, Yee J, Vijapurkar U, Canovatchel W, Meininger G. | Curr Med Res Opin. 2015 Nov;31(11):1993-2000. doi: 10.1185/03007995.2015.1082991. Epub 2015 Sep 28. | Select MA |  |  |
| Beneficial effect of lixisenatide after 76 weeks of treatment in patients with type 2 diabetes mellitus: A meta-analysis from the GetGoal programme. | Broglio F, Mannucci E, Napoli R, Nicolucci A, Purrello F, Nikonova E, Stager W, Trevisan R. | Diabetes Obes Metab. 2017 Feb;19(2):248-256. doi: 10.1111/dom.12810. Epub 2016 Dec 8. | Select MA |  |  |
| Cardiovascular effects of dapagliflozin in patients with type 2 diabetes and different risk categories: a meta-analysis. | Sonesson C, Johansson PA, Johnsson E, Gause-Nilsson I. | Cardiovasc Diabetol. 2016 Feb 19;15:37. doi: 10.1186/s12933-016-0356-y. | Select MA |  |  |
| Cardiovascular safety for once-weekly dulaglutide in type 2 diabetes: a pre-specified meta-analysis of prospectively adjudicated cardiovascular events. | Ferdinand KC, Botros FT, Atisso CM, Sager PT. | Cardiovasc Diabetol. 2016 Feb 24;15:38. doi: 10.1186/s12933-016-0355-z. | Select MA |  |  |
| Cardiovascular safety of empagliflozin in patients with type 2 diabetes: a meta-analysis of data from randomized placebo-controlled trials. | Salsali A, Kim G, Woerle HJ, Broedl UC, Hantel S. | Diabetes Obes Metab. 2016 Oct;18(10):1034-40. doi: 10.1111/dom.12734. Epub 2016 Aug 24. | Select MA |  |  |
| Clinical perspectives from the BEGIN and EDITION programmes: Trial-level meta-analyses outcomes with either degludec or glargine 300 U/mL vs glargine 100 U/mL in T2DM | Roussel R., Ritzel R., Boëlle-Le Corfec E., Balkau B., Rosenstock J. | Diabetes and Metabolism (2018) 44:5 (402-409). Date of Publication: 1 Nov 2018 | Select MA |  |  |
| Comparative effectiveness of exenatide once-weekly versus liraglutide in routine clinical practice: A retrospective multicentre study and meta-analysis of observational studies | Fadini G.P., Bonora B.M., Lapolla A., Fattor B., Morpurgo P.S., Simioni N., Avogaro A. | Diabetes, Obesity and Metabolism (2019). Date of Publication: 2019 | Select MA |  |  |
| Dapagliflozin-induced weight loss affects 24-week glycated haemoglobin and blood pressure levels. | SjÃ¶strÃ¶m CD, Hashemi M, Sugg J, Ptaszynska A, Johnsson E. | Diabetes Obes Metab. 2015 Aug;17(8):809-12. doi: 10.1111/dom.12500. Epub 2015 Jun 27. | Select MA |  |  |
| Dipeptidyl peptidase-4 inhibitors moderate the risk of genitourinary tract infections associated with sodium-glucose co-transporter-2 inhibitors. | Fadini GP, Bonora BM, Mayur S, Rigato M, Avogaro A. | Diabetes Obes Metab. 2018 Mar;20(3):740-744. doi: 10.1111/dom.13130. Epub 2017 Nov 15. | Select MA |  |  |
| Efficacy and Safety of Canagliflozin in Type 2 Diabetes Patients of Different Ethnicity. | Davidson JA, Aguilar R, Lavalle GonzÃ¡lez FJ, Trujillo A, Alba M, Vijapurkar U, Meininger G. | Ethn Dis. 2016 Apr 21;26(2):221-8. doi: 10.18865/ed.26.2.221. | Select MA |  |  |
| Efficacy and safety of lixisenatide in patients with type 2 diabetes and renal impairment | Hanefeld M., Arteaga J.M., Leiter L.A., Marchesini G., Nikonova E., Shestakova M., Stager W., Gómez-Huelgas R. | Diabetes, Obesity and Metabolism (2017) 19:11 (1594-1601). Date of Publication: 1 Nov 2017 | Select MA |  |  |
| Evaluation of unmet medical need among Japanese patients with type 2 diabetes mellitus and efficacy of Lixisenatide treatment among Asian type 2 diabetes mellitus patients. | Terauchi Y, Naito Y, Ikeda Y. | Diabetes Metab Syndr. 2016 Jan-Mar;10(1):23-8. doi: 10.1016/j.dsx.2015.08.008. Epub 2015 Aug 22. | Select MA |  |  |
| GLP-1 Receptor Agonists and Cardiovascular Disease: a Meta-Analysis of Recent Cardiac Outcome Trials. | Jia X, Alam M, Ye Y, Bajaj M, Birnbaum Y. | Cardiovasc Drugs Ther. 2018 Feb;32(1):65-72. doi: 10.1007/s10557-018-6773-2. | Select MA |  |  |
| Glycaemic control and hypoglycaemia benefits with insulin glargine 300 U/mL extend to people with type 2 diabetes and mild-to-moderate renal impairment | Javier Escalada F., Halimi S., Senior P.A., Bonnemaire M., Cali A.M.G., Melas-Melt L., Karalliedde J., Ritzel R.A. | Diabetes, Obesity and Metabolism (2018) 20:12 (2860-2868). Date of Publication: 1 Dec 2018 | Select MA |  |  |
| Lixisenatide as add-on treatment among patients with different Î²-cell function levels as assessed by HOMA-Î² index. | Bonadonna RC, Blonde L, Antsiferov M, Berria R, Gourdy P, Hatunic M, Mohan V, Horowitz M. | Diabetes Metab Res Rev. 2017 Sep;33(6). doi: 10.1002/dmrr.2897. Epub 2017 Jun 20. | Select MA |  |  |
| Risk of hypoglycaemia in people aged â‰¥65Â years receiving linagliptin: pooled data from 1489 individuals with type 2 diabetes mellitus. | Nauck M, Araki A, Hehnke U, Plat A, Clark D, Khunti K. | Int J Clin Pract. 2018 Oct;72(10):e13240. doi: 10.1111/ijcp.13240. Review. | Select MA |  |  |
| Safety and Tolerability of Empagliflozin in Patients with Type 2 Diabetes. | Kohler S, Salsali A, Hantel S, Kaspers S, Woerle HJ, Kim G, Broedl UC. | Clin Ther. 2016 Jun;38(6):1299-1313. doi: 10.1016/j.clinthera.2016.03.031. Epub 2016 Apr 13. | Select MA |  |  |
| Switching to insulin glargine 300 U/mL: Is duration of prior basal insulin therapy important? | Bonadonna RC, Renard E, Cheng A, Fritsche A, Cali A, Melas-Melt L, Umpierrez GE. | Diabetes Res Clin Pract. 2018 Aug;142:19-25. doi: 10.1016/j.diabres.2018.03.041. Epub 2018 Apr 9. | Select MA |  |  |
| The co-formulation of insulin degludec and insulin aspart lowers fasting plasma glucose and rates of confirmed and nocturnal hypoglycaemia, independent of baseline glycated haemoglobin levels, disease duration or body mass index: A pooled meta-analysis of phase III studies in patients with type 2 di... | HaluzÃ­k M, Fulcher G, Pieber TR, Bardtrum L, Tutkunkardas D, Rodbard HW. | Diabetes Obes Metab. 2018 Jul;20(7):1585-1592. doi: 10.1111/dom.13261. Epub 2018 Mar 25. | Select MA |  |  |
| Bayesian network meta-analysis (NMA) to assess relative efficacy of canagliflozin (CANA) versus glucagon-like peptide-1 (GLP-1) agonists in dual and triple therapy in patients with type 2 diabetes mellitus (T2DM) | Van Sanden S., Diels J., Guillon P., Nielsen A.T. | Value in Health (2015) 18:3 (A54). Date of Publication: May 2015 | NMA |  |  |
| Canagliflozin, dapagliflozin and empagliflozin monotherapy for treating type 2 diabetes: systematic review and economic evaluation. | Johnston R, Uthman O, Cummins E, Clar C, Royle P, Colquitt J, Tan BK, Clegg A, Shantikumar S, Court R, O'Hare JP, McGrane D, Holt T, Waugh N. | Health Technol Assess. 2017 Jan;21(2):1-218. doi: 10.3310/hta21020. Review. Erratum in: Health Technol Assess. 2018 Feb;21(2):219-220. | NMA |  |  |
| Comparison between sodium-glucose cotransporter 2 inhibitors and pioglitazone as additions to insulin therapy in type 2 diabetes patients: A systematic review with an indirect comparison meta-analysis. | Cho YK, Kim YJ, Kang YM, Lee SE, Park JY, Lee WJ, Jung CH. | J Diabetes Investig. 2018 Jul;9(4):882-892. doi: 10.1111/jdi.12787. Epub 2018 Jan 8. Review. | NMA |  |  |
| Evaluating the costs of glycemic response with canagliflozin versus dapagliflozin and empagliflozin as add-on to metformin in patients with type 2 diabetes mellitus in the United Arab Emirates. | Schubert A, Buchholt AT, El Khoury AC, Kamal A, Taieb V. | Curr Med Res Opin. 2017 Jun;33(6):1155-1163. doi: 10.1080/03007995.2017.1310091. Epub 2017 Apr 28. Review. | NMA |  |  |
| Hypoglycaemia when adding sulphonylurea to metformin: a systematic review and network meta-analysis. | Andersen SE, Christensen M. | Br J Clin Pharmacol. 2016 Nov;82(5):1291-1302. doi: 10.1111/bcp.13059. Epub 2016 Aug 3. Review. | NMA |  |  |
| Meta-Analysis of Effects of Sodium-Glucose Cotransporter 2 Inhibitors on Cardiovascular Outcomes and All-Cause Mortality Among Patients With Type 2 Diabetes Mellitus. | Tang H, Fang Z, Wang T, Cui W, Zhai S, Song Y. | Am J Cardiol. 2016 Dec 1;118(11):1774-1780. doi: 10.1016/j.amjcard.2016.08.061. Epub 2016 Aug 31. Review. | NMA |  |  |
| SGLT2 inhibitors and risk of cancer in type 2 diabetes: a systematic review and meta-analysis of randomised controlled trials. | Tang H, Dai Q, Shi W, Zhai S, Song Y, Han J. | Diabetologia. 2017 Oct;60(10):1862-1872. doi: 10.1007/s00125-017-4370-8. Epub 2017 Jul 19. Review. | NMA |  |  |
| Update in Cardiovascular Safety of Glucagon Like Peptide-1 Receptor Agonists in Patients With Type 2 Diabetes. A Mixed Treatment Comparison Meta-Analysis of Randomised Controlled Trials. | Al Yami MS, Alfayez OM, Alsheikh R. | Heart Lung Circ. 2018 Nov;27(11):1301-1309. doi: 10.1016/j.hlc.2018.03.018. Epub 2018 Mar 29. Review. | NMA |  |  |
| Urinary tract and genital infections in patients with type 2 diabetes treated with sodium-glucose co-transporter 2 inhibitors: A meta-analysis of randomized controlled trials. | Li D, Wang T, Shen S, Fang Z, Dong Y, Tang H. | Diabetes Obes Metab. 2017 Mar;19(3):348-355. doi: 10.1111/dom.12825. Epub 2016 Dec 19. | NMA |  |  |
| Individualizing treatment of hyperglycemia in type 2 diabetes | Fazel M.T., Pendergrass M.L. | Journal of Clinical Outcomes Management (2017) 24:1. Date of Publication: 1 Jan 2017 | Narrative |  |  |
| Blood glucose reduction by diabetic drugs with minimal hypoglycaemia risk for cardiovascular outcomes: Evidence from meta-regression analysis of randomized controlled trials. | Huang CJ, Wang WT, Sung SH, Chen CH, Lip GYH, Cheng HM, Chiang CE. | Diabetes Obes Metab. 2018 Sep;20(9):2131-2139. doi: 10.1111/dom.13342. Epub 2018 May 29. | Meta regression |  |  |
| Better glycaemic control and less hypoglycaemia with insulin glargine 300 U/mL vs glargine 100 U/mL: 1-year patient-level meta-analysis of the EDITION clinical studies in people with type 2 diabetes. | Ritzel R, Roussel R, Giaccari A, Vora J, Brulle-Wohlhueter C, Yki-JÃ¤rvinen H. | Diabetes Obes Metab. 2018 Mar;20(3):541-548. doi: 10.1111/dom.13105. Epub 2017 Oct 5. Review. | IPD MA |  |  |
| Patient-level meta-analysis of the EDITION 1, 2 and 3 studies: glycaemic control and hypoglycaemia with new insulin glargine 300 U/ml versus glargine 100 U/ml in people with type 2 diabetes. | Ritzel R, Roussel R, Bolli GB, Vinet L, Brulle-Wohlhueter C, Glezer S, Yki-JÃ¤rvinen H. | Diabetes Obes Metab. 2015 Sep;17(9):859-67. doi: 10.1111/dom.12485. Epub 2015 Jun 16. | IPD MA |  |  |
| Hypoglycaemic therapy in type 2 diabetes. Part I. Metformin is the only glucose-lowering drug known to prevent complications of diabetes. | [No authors listed] | Prescrire Int. 2015 Apr;24(159):103-6. Review. | Guidance |  |  |
| Cost effectiveness of dipeptidyl peptidase-4 inhibitors for type 2 diabetes. | Geng J, Yu H, Mao Y, Zhang P, Chen Y. | Pharmacoeconomics. 2015 Jun;33(6):581-97. doi: 10.1007/s40273-015-0266-y. Review. | Economic study |  |  |
| Saxagliptin in treatment of type 2 diabetes mellitus: A systematic review of pharmacoeconomic studies | Men P., Zhou J.-W., Tang H.-L., Zhai S.-D. | Chinese Pharmaceutical Journal (2016) 51:12 (1044-1048). Date of Publication: 22 Jun 2016 | Economic study |  |  |
| The place of DPP-4 inhibitors in the treatment algorithm of diabetes type 2: a systematic review of cost-effectiveness studies. | Baptista A, Teixeira I, Romano S, Carneiro AV, Perelman J. | Eur J Health Econ. 2017 Nov;18(8):937-965. doi: 10.1007/s10198-016-0837-7. Epub 2016 Oct 17. Review. | Economic study |  |  |
| Risk of heart failure with dipeptidyl peptidase-4 inhibitors in patients with type 2 diabetes mellitus: A meta-analysis of randomized controlled trials. | Kundu A, Sardar P, Ghosh S, Patel P, Chatterjee S, Meyer TE. | Int J Cardiol. 2016 Jun 1;212:203-5. doi: 10.1016/j.ijcard.2016.03.016. Epub 2016 Mar 19. No abstract available. | Correspondence |  |  |
| Cardiovascular safety of saxagliptin in patients with type 2 diabetes mellitus: A systematic review and meta-analysis | Mothe R.K., Ganji K., Esam H., Likhar N., Chidirala S.R., Sharma A., Jain P., Sirumalla Y., Dang A. | Value in Health (2016) 19:7 (A668). Date of Publication: 1 Nov 2016 | Conference abstract |  |  |
| Efficacy and safety of acarbose combined with insulin in treatment of type 2 diabetes mellitus: A meta-analysis of randomized controlled trials | Hu H., Zheng J., Chen X., Jiang D., Sun X., Qian Y., Zhang Y., Chen J. | International Journal of Clinical and Experimental Medicine (2017) 10:11 (15071-15079). Date of Publication: 30 Nov 2017 | Conference abstract |  |  |
| Efficacy and safety of albiglutide in the treatment of type II diabetes mellitus: A meta-analysis of randomized controlled trials | Esam H., Pagada A., Rai M.K. | Value in Health (2017) 20:5 (A166). Date of Publication: 1 May 2017 | Conference abstract |  |  |
| Efficacy and safety of antidiabetic drugs available on Brazilian public health system (SUS) - Regular insulin, NPH insulin, metformin, glibenclamide and gliclazide - In treatment of type 2 diabetes (T2DM) - Systematic review and meta-analysis | Alvares J., Araujo V.E., Izidoro J.B., Diniz L.M., Nascimento R.C., Silva M.R., Dias C.Z., Moreira D.P., Guerra Jr. A.A., Acurcio Fd. | Value in Health (2015) 18:7 (A862). Date of Publication: November 2015 | Conference abstract |  |  |
| Efficacy and safety of dulaglutide in the management of type 2 diabetes mellitus: A meta-analysis of randomized controlled trials | Kanukula R., Likhar N., Mothe R.K., Dang A. | Value in Health (2016) 19:3 (A198-A199). Date of Publication: May 2016 | Conference abstract |  |  |
| Safety and efficiency of SGLT 2 inhibitor combining with insulin in subjects with diabetes: Systematic review and meta-analysis of randomized controlled trials | Yang Y. | Endocrine Practice (2017) 23:1 (17A). Date of Publication: 1 Jan 2017 | Conference abstract |  |  |
| Safety assessment of canagliflozin for type 2 diabetes mellitus | Fan G., Han R., Zhang Y., Zhang Z., Liu Y., Wang D., He S., Chen Z. | International Journal of Clinical and Experimental Medicine (2016) 9:2 (2595-2612). Date of Publication: 29 Feb 2016 | Conference abstract |  |  |
| Management of dipeptidyl peptidase-4 inhibitor-associated angioedema in type 2 diabetes patients: A systematic review | Elsayed R., Walker A. | JACCP Journal of the American College of Clinical Pharmacy (2018) 1:2 (334-335). Date of Publication: 1 Dec 2018 | Conference abstract |  |  |
| Glucagon-like peptide-1 receptor agonists and risk of acute pancreatitis in patients with type 2 diabetes. | Storgaard H, Cold F, Gluud LL, VilsbÃ¸ll T, Knop FK. | Diabetes Obes Metab. 2017 Jun;19(6):906-908. doi: 10.1111/dom.12885. Epub 2017 Mar 17. Review. | Brief report |  |  |
| An Evaluation of the Clinical Therapeutic Effect of Lixisenatide in Type 2 Diabetes Patients: A Systematic Literature Review. | Okere AN, Montesdeoca J, Glasper A, Diaby V. | Curr Diabetes Rev. 2018;14(4):363-375. doi: 10.2174/1573399813666170724113240. Review. | Cannot access |  |  |
| Disparities in the Efficacy of Metformin in Combination with Dipeptidyl Peptidase-4 Inhibitor as Initial Treatment Stratified by Dosage and Ethnicity: A Meta-Analysis. | Cai X, Gao X, Yang W, Ji L. | Diabetes Technol Ther. 2018 Oct;20(10):704-714. doi: 10.1089/dia.2018.0124. Epub 2018 Aug 10. Review. | Cannot access |  |  |
| Effect of Glucagon-like Peptide-1 Receptor Agonists on All-cause Mortality and Cardiovascular Outcomes: A Meta-analysis. | Peterson SC, Barry AR. | Curr Diabetes Rev. 2018;14(3):273-279. doi: 10.2174/1573399813666170414101450. Review. | Cannot access |  |  |
| Efficacy and safety of piolitazone combined with metformin in type 2 diabetes mellitus: A meta-analysis | Gao Q., Liu X.-L. | Chinese Journal of Evidence-Based Medicine (2016) 16:10 (1148-1153). Date of Publication: 2016 | Cannot access |  |  |
| Impact of weight loss (WL) on health-related quality of life (HRQOL) among Latin American (LA) subjects with type 2 diabetes mellitus (T2DM) in phase 3 studies of canagliflozin (CANA) | Soares B., Akapame S., Cabrera P., Slee A., Traina S., Magno L. | Value in Health (2017) 20:9 (A921). Date of Publication: 1 Oct 2017 | Cannot access |  |  |
| Metformin improves survival in lung cancer patients with type 2 diabetes mellitus: A meta-analysis | Zeng S., Gan H.-X., Xu J.-X., Liu J.-Y. | Medicina Clinica (2018). Date of Publication: 2018 | Cannot access |  |  |
| Relationship of metformin with the risk of pancreatic cancer in patients with type 2 diabetes: A meta-analysis | Hu H., Fang Y., Zhou X., Gong L., Liu L., Wang W., Sun J., Zhai C., Pan H., Dong Y., Pan H. | Biomedical Research (India) (2017) 28:10 (4439-4444). Date of Publication: 2017 | Cannot access |  |  |
| Association Between Severe Hypoglycemia and Cardiovascular Disease Risk in Japanese Patients With Type 2 Diabetes. | Goto A, Goto M, Terauchi Y, Yamaguchi N, Noda M. | J Am Heart Assoc. 2016 Mar 9;5(3):e002875. doi: 10.1161/JAHA.115.002875. Review. Erratum in: J Am Heart Assoc. 2016 Jun;5(6). pii: e002075. doi: 10.1161/JAHA.116.002075. | 1 | 0 |  |
| Diabetes self-management education for adults with type 2 diabetes mellitus: A systematic review of the effect on glycemic control. | Chrvala CA, Sherr D, Lipman RD. | Patient Educ Couns. 2016 Jun;99(6):926-43. doi: 10.1016/j.pec.2015.11.003. Epub 2015 Nov 22. Review. | 1 | 0 |  |
| Efficacy and effectiveness of screen and treat policies in prevention of type 2 diabetes: systematic review and meta-analysis of screening tests and interventions. | Barry E, Roberts S, Oke J, Vijayaraghavan S, Normansell R, Greenhalgh T. | BMJ. 2017 Jan 4;356:i6538. doi: 10.1136/bmj.i6538. Review. | 1 | 0 |  |
| Glucose regulation, cognition, and brain MRI in type 2 diabetes: A systematic review | Geijselaers S.L.C., Sep S.J.S., Stehouwer C.D.A., Biessels G.J. | The Lancet Diabetes and Endocrinology (2015) 3:1 (75-89). Date of Publication: 1 Jan 2015 | 1 | 0 |  |
| Observational studies with type 2 diabetes mellitus treatments in Spain: A systematic literature review | Díaz S., Dilla T., Reviriego J. | Endocrinologia, Diabetes y Nutricion (2018). Date of Publication: 2018 | 1 | 0 |  |
| SCORE-IT (Selecting Core Outcomes for Randomised Effectiveness trials In Type 2 diabetes): a systematic review of registered trials. | Harman NL, James R, Wilding J, Williamson PR; SCORE-IT study team.. | Trials. 2017 Dec 15;18(1):597. doi: 10.1186/s13063-017-2317-5. Review. | 1 | 0 |  |
| A Systematic Review of Patient-Reported Satisfaction with Oral Medication Therapy in Patients with Type 2 Diabetes. | Wang Y, Perri M 3rd. | Value Health. 2018 Nov;21(11):1346-1353. doi: 10.1016/j.jval.2018.05.001. Epub 2018 Jul 17. Review. | 1 | 0 |  |
| Adverse cardiovascular outcomes between insulin-treated and non-insulin treated diabetic patients after percutaneous coronary intervention: a systematic review and meta-analysis. | Bundhun PK, Li N, Chen MH. | Cardiovasc Diabetol. 2015 Oct 7;14:135. doi: 10.1186/s12933-015-0300-6. Review. | 1 | 1 | Type 1+2 DM |
| Acarbose Monotherapy and Type 2 Diabetes Prevention in Eastern and Western Prediabetes: An Ethnicity-specific Meta-analysis. | Hu R, Li Y, Lv Q, Wu T, Tong N. | Clin Ther. 2015 Aug;37(8):1798-812. doi: 10.1016/j.clinthera.2015.05.504. Epub 2015 Jun 26. Review. | 1 | 1 | Prediabetes |
| Prognostic role of metformin intake in diabetic patients with colorectal cancer: An updated qualitative evidence of cohort studies. | Du L, Wang M, Kang Y, Li B, Guo M, Cheng Z, Bi C. | Oncotarget. 2017 Apr 18;8(16):26448-26459. doi: 10.18632/oncotarget.14688. | 1 | 1 | DM not specific |
| Efficacy and safety of oral insulin compared to subcutaneous insulin: a systematic review and meta-analysis. | Akbari V, Hendijani F, Feizi A, Varshosaz J, Fakhari Z, Morshedi S, Mostafavi SA. | J Endocrinol Invest. 2016 Feb;39(2):215-25. doi: 10.1007/s40618-015-0326-3. Epub 2015 Jun 24. Review. | 1 | 1 | Type 1+2 DM |
| Clinical efficacy and safety of insulin aspart compared with regular human insulin in patients with type 1 and type 2 diabetes: a systematic review and meta-analysis. | Wojciechowski P, Niemczyk-Szechowska P, OlewiÅ„ska E, Jaros P, Mierzejewska B, SkarÅ¼yÅ„ska-Duk J, MaÅ‚ecki MT, RyÅ› P. | Pol Arch Med Wewn. 2015;125(3):141-51. Epub 2015 Jan 30. Review. Erratum in: Pol Arch Med Wewn. 2015;125(4):308. | 1 | 1 | TYPE 1+2 DM |
| Comparative safety and efficacy of insulin degludec with insulin glargine in type 2 and type 1 diabetes: a meta-analysis of randomized controlled trials. | Zhang XW, Zhang XL, Xu B, Kang LN. | Acta Diabetol. 2018 May;55(5):429-441. doi: 10.1007/s00592-018-1107-1. Epub 2018 Feb 8. Review. | 1 | 1 | TYPE 1+2 DM |
| Drug-related risk of severe hypoglycaemia in observational studies: a systematic review and meta-analysis. | Czech M, Rdzanek E, PawÄ™ska J, Adamowicz-Sidor O, Niewada M, Jakubczyk M. | BMC Endocr Disord. 2015 Oct 12;15:57. doi: 10.1186/s12902-015-0052-z. Review. | 1 | 1 | TYPE 1+2 DM |
| Association between metformin therapy and incidence, recurrence and mortality of prostate cancer: evidence from a meta-analysis. | Deng D, Yang Y, Tang X, Skrip L, Qiu J, Wang Y, Zhang F. | Diabetes Metab Res Rev. 2015 Sep;31(6):595-602. doi: 10.1002/dmrr.2645. Epub 2015 Apr 20. | 1 | 1 | DM not specifically mentioned |
| Pioglitazone and cardiovascular outcomes in patients with insulin resistance, pre-diabetes and type 2 diabetes: a systematic review and meta-analysis. | Liao HW, Saver JL, Wu YL, Chen TH, Lee M, Ovbiagele B. | BMJ Open. 2017 Jan 5;7(1):e013927. doi: 10.1136/bmjopen-2016-013927. Review. | 1 | 1 | Pre-DM |
| Antidiabetic potential of Musa spp. inflorescence: a systematic review | de Oliveira Vilhena R., Fachi M.M., Marson B.M., Dias B.L., Pontes F.L.D., Tonin F.S., Pontarolo R. | Journal of Pharmacy and Pharmacology (2018) 70:12 (1583-1595). Date of Publication: 1 Dec 2018 | 1 | 1 | In vivo and in vitro |
| Effect of hypoglycemic agents on survival outcomes of lung cancer patients with diabetes mellitus: A meta-analysis. | Xin WX, Fang L, Fang QL, Zheng XW, Ding HY, Huang P. | Medicine (Baltimore). 2018 Mar;97(9):e0035. doi: 10.1097/MD.0000000000010035. | 1 | 1 | Type 1+2 DM |
| Metformin use improves survival of diabetic liver cancer patients: systematic review and meta-analysis. | Ma SJ, Zheng YX, Zhou PC, Xiao YN, Tan HZ. | Oncotarget. 2016 Oct 4;7(40):66202-66211. doi: 10.18632/oncotarget.11033. Review. | 1 | 1 | Mixed population |
| Comparative effectiveness of continuous subcutaneous insulin infusion using insulin analogs and multiple daily injections in pregnant women with diabetes mellitus: a systematic review and meta-analysis. | Ranasinghe PD, Maruthur NM, Nicholson WK, Yeh HC, Brown T, Suh Y, Wilson LM, Nannes EB, Berger Z, Bass EB, Golden SH. | J Womens Health (Larchmt). 2015 Mar;24(3):237-49. doi: 10.1089/jwh.2014.4939. Epub 2015 Feb 25. Review. | 1 | 1 | Type 1+2 DM |
| Effectiveness of insulin glargine U-300 versus insulin glargine U-100 on nocturnal hypoglycemia and glycemic control in type 1 and type 2 diabetes: a systematic review and meta-analysis | Díez-Fernández A., Cavero-Redondo I., Moreno-Fernández J., Pozuelo-Carrascosa D.P., Garrido-Miguel M., Martínez-Vizcaíno V. | Acta Diabetologica (2019) 56:3 (355-364). Date of Publication: 6 Mar 2019 | 1 | 1 | Type 1+2 DM |
| Efficacy, safety, and patient acceptability of Technosphere inhaled insulin for people with diabetes: a systematic review and meta-analysis. | Pittas AG, Westcott GP, Balk EM. | Lancet Diabetes Endocrinol. 2015 Nov;3(11):886-94. doi: 10.1016/S2213-8587(15)00280-6. Epub 2015 Sep 1. Review. | 1 | 1 | Type 1+2 DM |
| Long-term Sustainability of Diabetes Prevention Approaches: A Systematic Review and Meta-analysis of Randomized Clinical Trials. | Haw JS, Galaviz KI, Straus AN, Kowalski AJ, Magee MJ, Weber MB, Wei J, Narayan KMV, Ali MK. | JAMA Intern Med. 2017 Dec 1;177(12):1808-1817. doi: 10.1001/jamainternmed.2017.6040. Review. | 1 | 1 | Type 1+2 DM |
| MANAGEMENT OF DIABETES DURING AIR TRAVEL: A SYSTEMATIC LITERATURE REVIEW OF CURRENT RECOMMENDATIONS AND THEIR SUPPORTING EVIDENCE. | Pavela J, Suresh R, Blue RS, Mathers CH, Belalcazar LM. | Endocr Pract. 2018 Feb;24(2):205-219. doi: 10.4158/EP171954.RA. Review. | 1 | 1 | Type 1+2 DM |
| Safety and efficiency of SGLT2 inhibitor combining with insulin in subjects with diabetes | Yang Y., Chen S., Pan H., Zou Y., Wang B., Wang G., Zhu H. | Medicine (United States) (2017) 96:21 Article Number: e6944. Date of Publication: 1 May 2017 | 1 | 1 | Type 1+2 DM |
| SGLT2 Inhibitors: A Systematic Review of Diabetic Ketoacidosis and Related Risk Factors in the Primary Literature. | Burke KR, Schumacher CA, Harpe SE. | Pharmacotherapy. 2017 Feb;37(2):187-194. doi: 10.1002/phar.1881. Epub 2017 Jan 16. Review. | 1 | 1 | Type 1+2 DM |
| The effect of glucagon-like peptide 1 and glucagon-like peptide 1 receptor agonists on energy expenditure: A systematic review and meta-analysis. | Maciel MG, Beserra BTS, Oliveira FCB, Ribeiro CM, Coelho MS, Neves FAR, Amato AA. | Diabetes Res Clin Pract. 2018 Aug;142:222-235. doi: 10.1016/j.diabres.2018.05.034. Epub 2018 May 30. Review. | 1 | 1 | With and without T2DM |
| Effect of metformin on exercise capacity: A meta-analysis. | Das S, Behera SK, Srinivasan A, Xavier AS, Selvarajan S, Kamalanathan S, Sahoo JP, Nair NS. | Diabetes Res Clin Pract. 2018 Oct;144:270-278. doi: 10.1016/j.diabres.2018.08.022. Epub 2018 Sep 12. | 1 | 1 | Healthy volunteers and T2DM |
| Creatine Supplementation and glycemic control: a systematic review. | Pinto CL, Botelho PB, Pimentel GD, Campos-Ferraz PL, Mota JF. | Amino Acids. 2016 Sep;48(9):2103-29. doi: 10.1007/s00726-016-2277-1. Epub 2016 Jun 15. Review. | 1 | 1 | 2 of 19 articles were T2DM |

1 denotes “meets criteria”; 2 = “does not meet criteria”; NMA = network meta-analysis; IPD MA = individual patient data meta analysis

# Studies Excluded Due to No Publicly Available Protocol

| Title | Study Authors | Reference |
| --- | --- | --- |
| A nomogram to estimate the proportion of patients at hemoglobin A1c target <7% with noninsulin antidiabetic drugs in type 2 diabetes: A systematic review of 137 randomized controlled trials with 39 845 patients | Esposito K., Chiodini P., Ceriello A., Giugliano D. | Acta Diabetologica (2014) 51:2 (305-311). Date of Publication: April 2014 |
| Combinational therapy with metformin and sodium-glucose cotransporter inhibitors in management of type 2 diabetes: Systematic review and meta-analyses | Zhang Q., Dou J., Lu J. | Diabetes Research and Clinical Practice (2014) 105:3 (313-321). Date of Publication: 2014 |
| Metformin in patients with type 2 diabetes and kidney disease a systematic review | Inzucchi S.E., Lipska K.J., Mayo H., Bailey C.J., McGuire D.K. | JAMA - Journal of the American Medical Association (2014) 312:24 (2668-2675). Date of Publication: 24 Dec 2014 |
| A nomogram to estimate the HbA1c response to different DPP-4 inhibitors in type 2 diabetes: a systematic review and meta-analysis of 98 trials with 24â€…163 patients. | Esposito K, Chiodini P, Maiorino MI, Capuano A, Cozzolino D, Petrizzo M, Bellastella G, Giugliano D. | BMJ Open. 2015 Feb 16;5(2):e005892. doi: 10.1136/bmjopen-2014-005892. Review. |
| Anti-atherosclerotic effects of the glucagon-like peptide-1 (GLP-1) based therapies in patients with type 2 Diabetes Mellitus: A meta-analysis. | Song X, Jia H, Jiang Y, Wang L, Zhang Y, Mu Y, Liu Y. | Sci Rep. 2015 Jun 26;5:10202. doi: 10.1038/srep10202. |
| Assessing the efficacy and safety of combined DPP-4 inhibitor and insulin treatment in patients with type 2 diabetes: a meta-analysis. | Chen C, Yu Q, Zhang S, Yang P, Wang CY. | Int J Clin Exp Pathol. 2015 Nov 1;8(11):14141-50. eCollection 2015. Review. |
| Barriers and facilitators to starting insulin in patients with type 2 diabetes: a systematic review. | Ng CJ, Lai PS, Lee YK, Azmi SA, Teo CH. | Int J Clin Pract. 2015 Oct;69(10):1050-70. doi: 10.1111/ijcp.12691. Epub 2015 Jul 6. Review. |
| Biphasic vs basal bolus insulin regimen in Type¬†2 diabetes: a systematic review and meta-analysis of randomized controlled trials. | Wang C, Mamza J, Idris I. | Diabet Med. 2015 May;32(5):585-94. doi: 10.1111/dme.12694. Epub 2015 Feb 13. Review. |
| Comparison of glucose lowering effect of metformin and acarbose in type 2 diabetes mellitus: A meta-analysis | Gu S., Shi J., Tang Z., Sawhney M., Hu H., Shi L., Fonseca V., Dong H. | PLoS ONE (2015) 10:5 Article Number: e0126704. Date of Publication: 11 May 2015 |
| Comparisons of the efficacy of glucose control, lipid profile, and Î²-cell function between DPP-4 inhibitors and AGI treatment in type 2 diabetes patients: a meta-analysis. | Cai X, Yang W, Zhou L, Zhang S, Han X, Ji L. | Endocrine. 2015 Dec;50(3):590-7. doi: 10.1007/s12020-015-0653-3. Epub 2015 Jun 6. |
| Effect and cardiovascular safety of adding rosiglitazone to insulin therapy in type 2 diabetes: A meta-analysis | Lu Y., Ma D., Xu W., Shao S., Yu X. | Journal of Diabetes Investigation (2015) 6:1 (78-86). Date of Publication: 1 Jan 2015 |
| Effect of Intensive Versus Standard Blood Glucose Control in Patients With Type 2 Diabetes Mellitus in Different Regions of the World: Systematic Review and Meta-analysis of Randomized Controlled Trials. | Sardar P, Udell JA, Chatterjee S, Bansilal S, Mukherjee D, Farkouh ME. | J Am Heart Assoc. 2015 May 5;4(5). pii: e001577. doi: 10.1161/JAHA.114.001577. Review. |
| Effectiveness and safety of glimepiride and iDPP4, associated with metformin in second line pharmacotherapy of type 2 diabetes mellitus: systematic review and meta-analysis. | Amate JM, Lopez-Cuadrado T, Almendro N, Bouza C, Saz-Parkinson Z, Rivas-Ruiz R, Gonzalez-Canudas J. | Int J Clin Pract. 2015 Mar;69(3):292-304. doi: 10.1111/ijcp.12605. Epub 2015 Feb 16. Review. |
| Effects and safety of canagliflozin in the treatment of type 2 diabetes mellitus: A systematic review and meta-analysis | Zhang S., Cao G. | Pharmacotherapy (2015) 35:11 (e299). Date of Publication: November 2015 |
| Effects of intensive glycemic control in ocular complications in patients with type 2 diabetes: a meta-analysis of randomized clinical trials. | Zhang X, Zhao J, Zhao T, Liu H. | Endocrine. 2015 May;49(1):78-89. doi: 10.1007/s12020-014-0459-8. Epub 2014 Oct 30. |
| Effects of sulfonylureas on lipids in type 2 diabetes mellitus: a meta-analysis of randomized controlled trials. | Chen YH, Du L, Geng XY, Peng YL, Shen JN, Zhang YG, Liu GJ, Sun X. | J Evid Based Med. 2015 Aug;8(3):134-48. doi: 10.1111/jebm.12157. |
| Effects of thiazolidinedione therapy on inflammatory markers of type 2 diabetes: a meta-analysis of randomized controlled trials. | Chen R, Yan J, Liu P, Wang Z. | PLoS One. 2015 Apr 21;10(4):e0123703. doi: 10.1371/journal.pone.0123703. eCollection 2015. |
| Efficacy and safety of an extended-release metformin formulation in type-2 diabetes mellitus treatment: A systematic review | Fujii R.K., Restrepo M., Fernandes R.A., Haas L., Pepe C., Turatti L.A., Junqueira M. | Value in Health (2015) 18:3 (A56). Date of Publication: May 2015 |
| Efficacy and safety of canagliflozin among patients with type 2 diabetes mellitus: A systematic review and meta-analysis | Kaur K., Likhar N., Dang A. | Value in Health (2015) 18:3 (A56-A57). Date of Publication: May 2015 |
| Efficacy and safety of Flos Abelmoschus manihot (Malvaceae) on type 2 diabetic nephropathy: A systematic review | Chen Y.-Z., Gong Z.-X., Cai G.-Y., Gao Q., Chen X.-M., Tang L., Wei R.-B., Zhou J.-H. | Chinese Journal of Integrative Medicine (2015) 21:6 (464-472). Date of Publication: 9 Dec 2015 |
| Efficacy and safety of once-weekly glucagon-like peptide 1 receptor agonists for the management of type 2 diabetes: a systematic review and meta-analysis of randomized controlled trials. | Karagiannis T, Liakos A, Bekiari E, Athanasiadou E, Paschos P, Vasilakou D, Mainou M, Rika M, Boura P, Matthews DR, Tsapas A. | Diabetes Obes Metab. 2015 Nov;17(11):1065-74. doi: 10.1111/dom.12541. Epub 2015 Sep 23. Review. |
| Efficacy and safety of sitagliptin compared with sulfonylurea therapy in patients with type 2 diabetes showing inadequately controlled glycosylated hemoglobin with metformin monotherapy: A Meta-Analysis | Hou L., Zhao T., Liu Y., Zhang Y. | Experimental and Therapeutic Medicine (2015) 9:4 (1528-1536). Date of Publication: 2015 |
| Efficacy and safety of sodium-glucose cotransporter 2 inhibitors in type 2 diabetes: a meta-analysis of randomized controlled trials for 1 to 2years. | Liu XY, Zhang N, Chen R, Zhao JG, Yu P. | J Diabetes Complications. 2015 Nov-Dec;29(8):1295-303. doi: 10.1016/j.jdiacomp.2015.07.011. Epub 2015 Jul 21. |
| Efficacy and tolerability of canagliflozin as add-on to metformin in the treatment of type 2 diabetes mellitus: a meta-analysis. | Yang T, Lu M, Ma L, Zhou Y, Cui Y. | Eur J Clin Pharmacol. 2015 Nov;71(11):1325-32. doi: 10.1007/s00228-015-1923-y. Epub 2015 Aug 19. |
| Efficacy of dipeptidyl-peptidase-4 inhibitors and impact on Œ≤-cell function in Asian and Caucasian type 2 diabetes mellitus patients: A meta-analysis. | Cai X, Han X, Luo Y, Ji L. | J Diabetes. 2015 May;7(3):347-59. doi: 10.1111/1753-0407.12196. Epub 2014 Sep 10. |
| Glucagon-like peptide 1 receptor agonist therapy is more efficacious than insulin glargine for poorly controlled type 2 diabetes: A systematic review and meta-analysis. | Liu FP, Dong JJ, Yang Q, Xue XZ, Ren ZF, Gan YZ, Liao L. | J Diabetes. 2015 May;7(3):322-8. doi: 10.1111/1753-0407.12200. Epub 2014 Sep 10. Review. |
| Glucose-lowering drugs or strategies and cardiovascular outcomes in patients with or at risk for type 2 diabetes: a meta-analysis of randomised controlled trials. | Udell JA, Cavender MA, Bhatt DL, Chatterjee S, Farkouh ME, Scirica BM. | Lancet Diabetes Endocrinol. 2015 May;3(5):356-66. doi: 10.1016/S2213-8587(15)00044-3. Epub 2015 Mar 17. |
| HbA1c below 7% as the goal of glucose control fails to maximize the cardiovascular benefits: a meta-analysis. | Wang P, Huang R, Lu S, Xia W, Sun H, Sun J, Cai R, Wang S. | Cardiovasc Diabetol. 2015 Sep 22;14:124. doi: 10.1186/s12933-015-0285-1. |
| Incidence of hypoglycemia in patients with type 2 diabetes treated with gliclazide versus DPP-4 inhibitors during Ramadan: A meta-analytical approach. | Mbanya JC, Al-Sifri S, Abdel-Rahim A, Satman I. | Diabetes Res Clin Pract. 2015 Aug;109(2):226-32. doi: 10.1016/j.diabres.2015.04.030. Epub 2015 May 8. Review. |
| Incretin-based therapies are associated with acute pancreatitis: Meta-analysis of large randomized controlled trials. | Roshanov PS, Dennis BB. | Diabetes Res Clin Pract. 2015 Dec;110(3):e13-7. doi: 10.1016/j.diabres.2015.10.014. Epub 2015 Oct 23. |
| Metformin and lung cancer risk of patients with type 2 diabetes mellitus: A meta-analysis | Zhu N., Zhang Y., Gong Y., He J., Chen X. | Biomedical Reports (2015) 3:2 (235-241). Date of Publication: 2015 |
| Metformin as a risk factor of pathogenesis of colorectal cancer in type 2 diabetes patients: A metaanalysis | Yan Y., Tang S.-H., Huang Q.-Y., Tang H.-J. | Medical Journal of Chinese People's Liberation Army (2015) 40:7 (582-586). Date of Publication: 25 Jul 2015 |
| Metformin association with lower prostate cancer recurrence in type 2 diabetes: a systematic review and meta-analysis. | Hwang IC, Park SM, Shin D, Ahn HY, Rieken M, Shariat SF. | Asian Pac J Cancer Prev. 2015;16(2):595-600. Review. |
| Pharmacologic Therapy of Diabetes and Overall Cancer Risk and Mortality: A Meta-Analysis of 265 Studies. | Wu L, Zhu J, Prokop LJ, Murad MH. | Sci Rep. 2015 Jun 15;5:10147. doi: 10.1038/srep10147. Review. |
| Predictors of response in initial users of metformin and sulphonylurea derivatives: a systematic review. | Martono DP, Lub R, Lambers Heerspink HJ, Hak E, Wilffert B, Denig P. | Diabet Med. 2015 Jul;32(7):853-64. doi: 10.1111/dme.12688. Epub 2015 Feb 9. Review. |
| Prevalence and Incidence of Hypoglycaemia in 532,542 People with Type 2 Diabetes on Oral Therapies and Insulin: A Systematic Review and Meta-Analysis of Population Based Studies. | Edridge CL, Dunkley AJ, Bodicoat DH, Rose TC, Gray LJ, Davies MJ, Khunti K. | PLoS One. 2015 Jun 10;10(6):e0126427. doi: 10.1371/journal.pone.0126427. eCollection 2015. Review. |
| Systematic review and meta-analysis of randomized clinical trials comparing efficacy and safety outcomes of insulin glargine with NPH insulin, premixed insulin preparations or with insulin detemir in type 2 diabetes mellitus. | Rys P, Wojciechowski P, Rogoz-Sitek A, Niesyczy≈Ñski G, Lis J, Syta A, Malecki MT. | Acta Diabetol. 2015 Aug;52(4):649-62. doi: 10.1007/s00592-014-0698-4. Epub 2015 Jan 14. Review. |
| Systematic review and meta-analysis of the efficacy and hypoglycemic safety of gliclazide versus other insulinotropic agents. | Chan SP, Colagiuri S. | Diabetes Res Clin Pract. 2015 Oct;110(1):75-81. doi: 10.1016/j.diabres.2015.07.002. Epub 2015 Jul 9. Review. |
| The efficacy and safety of DPP4 inhibitors compared to sulfonylureas as add-on therapy to metformin in patients with Type 2 diabetes: A systematic review and meta-analysis. | Mishriky BM, Cummings DM, Tanenberg RJ. | Diabetes Res Clin Pract. 2015 Aug;109(2):378-88. doi: 10.1016/j.diabres.2015.05.025. Epub 2015 May 14. Review. |
| Using real-world data to evaluate the association of incretin-based therapies with risk of acute pancreatitis: a meta-analysis of 1,324,515 patients from observational studies. | Wang T, Wang F, Gou Z, Tang H, Li C, Shi L, Zhai S. | Diabetes Obes Metab. 2015 Jan;17(1):32-41. doi: 10.1111/dom.12386. Epub 2014 Oct 6. |
| A systematic review of the benefits and harms of dipeptidyl peptidase-4 inhibitor for chronic kidney disease. | Patel S, Gohel K, Patel BG. | Curr Diabetes Rev. 2016;12(3):211-22. Review. |
| Addition of dipeptidyl peptidase-4 inhibitors to sulphonylureas and risk of hypoglycaemia: systematic review and meta-analysis. | Salvo F, Moore N, Arnaud M, Robinson P, Raschi E, De Ponti F, B√©gaud B, Pariente A. | BMJ. 2016 May 3;353:i2231. doi: 10.1136/bmj.i2231. Review. |
| Alpha-glucosidase inhibitors and hepatotoxicity in type 2 diabetes: a systematic review and meta-analysis. | Zhang L, Chen Q, Li L, Kwong JS, Jia P, Zhao P, Wang W, Zhou X, Zhang M, Sun X. | Sci Rep. 2016 Sep 6;6:32649. doi: 10.1038/srep32649. Review. |
| An overview of the effect of sodium glucose cotransporter 2 inhibitor monotherapy on glycemic and other clinical laboratory parameters in type 2 diabetes patients | Wang Y., Hu X., Li X., Wang Z. | Therapeutics and Clinical Risk Management (2016) 12 (1113-1131). Date of Publication: 15 Jul 2016 |
| Association between metformin and vitamin B(12) deficiency in patients with type 2 diabetes: A systematic review and meta-analysis. | Chapman LE, Darling AL, Brown JE. | Diabetes Metab. 2016 Nov;42(5):316-327. doi: 10.1016/j.diabet.2016.03.008. Epub 2016 Apr 26. Review. |
| Bi-directional interaction between hypoglycaemia and cognitive impairment in elderly patients treated with glucose-lowering agents: a systematic review and meta-analysis. | Mattishent K, Loke YK. | Diabetes Obes Metab. 2016 Feb;18(2):135-41. doi: 10.1111/dom.12587. Epub 2015 Dec 8. Review. |
| Cardiovascular and non-cardiovascular safety of dipeptidyl peptidase-4 inhibition: a meta-analysis of randomized controlled cardiovascular outcome trials. | Abbas AS, Dehbi HM, Ray KK. | Diabetes Obes Metab. 2016 Mar;18(3):295-9. doi: 10.1111/dom.12595. Epub 2015 Dec 23. |
| Cardiovascular Effect of Incretin-Based Therapy in Patients with Type 2 Diabetes Mellitus: Systematic Review and Meta-Analysis. | Kim JY, Yang S, Lee JI, Chang MJ. | PLoS One. 2016 Apr 14;11(4):e0153502. doi: 10.1371/journal.pone.0153502. eCollection 2016. Review. Erratum in: PLoS One. 2018 Jan 19;13(1):e0191744. |
| Cardiovascular risk associated with the use of glitazones, metformin and sufonylureas: meta-analysis of published observational studies. | Pladevall M, Riera-Guardia N, Margulis AV, Varas-Lorenzo C, Calingaert B, Perez-Gutthann S. | BMC Cardiovasc Disord. 2016 Jan 15;16:14. doi: 10.1186/s12872-016-0187-5. |
| Colon neoplasia in patients with type 2 diabetes on metformin: A meta-analysis. | Rokkas T, Portincasa P. | Eur J Intern Med. 2016 Sep;33:60-6. doi: 10.1016/j.ejim.2016.05.027. Epub 2016 Jun 16. |
| Combined use of dipeptidyl peptidase-4 inhibitors and metformin reduces blood sugar level and improves pancreatic islet β cell function in the treatment of type 2 diabetes mellitus: A meta-analysis | Cheng W., Pan Y., Xu Q. | International Journal of Clinical and Experimental Medicine (2016) 9:11 (20624-20632) Article Number: IJCEM0034312. Date of Publication: 20 Dec 2016 |
| Comparative efficacy of anti-diabetic agents on nonalcoholic fatty liver disease in patients with type 2 diabetes mellitus: a systematic review and meta-analysis of randomized and non-randomized studies. | Tang W, Xu Q, Hong T, Tong G, Feng W, Shen S, Bi Y, Zhu D. | Diabetes Metab Res Rev. 2016 Feb;32(2):200-16. doi: 10.1002/dmrr.2713. Epub 2015 Nov 1. Review. |
| Comparative studies of dipeptidyl peptidase 4 inhibitor vs sulphonylurea among Muslim Type 2 diabetes patients who fast in the month of Ramadan: A systematic review and meta-analysis. | Loh HH, Yee A, Loh HS, Sukor N, Kamaruddin NA. | Prim Care Diabetes. 2016 Jun;10(3):210-9. doi: 10.1016/j.pcd.2015.09.001. Epub 2015 Oct 5. Review. |
| Comparing the clinical outcomes between insulin-treated and non-insulin-treated patients with type 2 diabetes mellitus after coronary artery bypass surgery: A systematic review and meta-analysis | Munnee K., Bundhun P.K., Quan H., Tang Z. | Medicine (United States) (2016) 95:10 Article Number: e3006. Date of Publication: 4 Mar 2016 |
| Diabetes Medications as Monotherapy or Metformin-Based Combination Therapy for Type 2 Diabetes: A Systematic Review and Meta-analysis. | Maruthur NM, Tseng E, Hutfless S, Wilson LM, Suarez-Cuervo C, Berger Z, Chu Y, Iyoha E, Segal JB, Bolen S. | Ann Intern Med. 2016 Jun 7;164(11):740-51. doi: 10.7326/M15-2650. Epub 2016 Apr 19. Review. |
| Differential HbA1c response in the placebo arm of DPP-4 inhibitor clinical trials conducted in China compared to other countries: a systematic review and meta-analysis. | He L, Liu S, Shan C, Tu Y, Li Z, Zhang XD. | BMC Pharmacol Toxicol. 2016 Sep 7;17(1):40. doi: 10.1186/s40360-016-0084-7. Review. |
| Dipeptidyl peptidase-4 inhibitors and fracture risk: an updated meta-analysis of randomized clinical trials. | Fu J, Zhu J, Hao Y, Guo C, Zhou Z. | Sci Rep. 2016 Jul 7;6:29104. doi: 10.1038/srep29104. |
| Dipeptidyl peptidase-4 inhibitors and risk of heart failure in type 2 diabetes: systematic review and meta-analysis of randomised and observational studies. | Li L, Li S, Deng K, Liu J, Vandvik PO, Zhao P, Zhang L, Shen J, Bala MM, Sohani ZN, Wong E, Busse JW, Ebrahim S, Malaga G, Rios LP, Wang Y, Chen Q, Guyatt GH, Sun X. | BMJ. 2016 Feb 17;352:i610. doi: 10.1136/bmj.i610. Review. |
| DPP-4 inhibitor therapy and bone fractures in people with Type 2 diabetes - A systematic review and meta-analysis. | Mamza J, Marlin C, Wang C, Chokkalingam K, Idris I. | Diabetes Res Clin Pract. 2016 Jun;116:288-98. doi: 10.1016/j.diabres.2016.04.029. Epub 2016 Apr 30. Review. |
| DPP-4 inhibitors and risk of infections: a meta-analysis of randomized controlled trials. | Yang W, Cai X, Han X, Ji L. | Diabetes Metab Res Rev. 2016 May;32(4):391-404. doi: 10.1002/dmrr.2723. Epub 2015 Nov 2. |
| Effectiveness of insulin glargine in type 2 diabetes mellitus patients failing glycaemic control with premixed insulin: Adriatic countries data meta-analysis. | Cigrovski Berkovic M, Petrovski G, Grulovic N. | Acta Diabetol. 2016 Oct;53(5):709-15. doi: 10.1007/s00592-016-0861-1. Epub 2016 Apr 21. |
| Effects of Dipeptidyl Peptidase 4 Inhibitors and Sodium-Glucose Linked coTransporter-2 Inhibitors on cardiovascular events in patients with type 2 diabetes mellitus: A meta-analysis. | Savarese G, D'Amore C, Federici M, De Martino F, Dellegrottaglie S, Marciano C, Ferrazzano F, Losco T, Lund LH, Trimarco B, Rosano GM, Perrone-Filardi P. | Int J Cardiol. 2016 Oct 1;220:595-601. doi: 10.1016/j.ijcard.2016.06.208. Epub 2016 Jun 27. |
| Effects of dipeptidyl peptidase-4 inhibitors on blood pressure in patients with type 2 diabetes: A systematic review and meta-analysis. | Zhang X, Zhao Q. | J Hypertens. 2016 Feb;34(2):167-75. doi: 10.1097/HJH.0000000000000782. Review. |
| Effects of glucose-lowering and multifactorial interventions on cardiovascular and mortality outcomes: a meta-analysis of randomized control trials. | Seidu S, Achana FA, Gray LJ, Davies MJ, Khunti K. | Diabet Med. 2016 Mar;33(3):280-9. doi: 10.1111/dme.12885. Epub 2015 Sep 8. Review. |
| Effects of intensive glucose lowering in treatment of type 2 diabetes mellitus on cardiovascular outcomes: A meta-analysis of data from 58,160 patients in 13 randomized controlled trials. | Fang HJ, Zhou YH, Tian YJ, Du HY, Sun YX, Zhong LY. | Int J Cardiol. 2016 Sep 1;218:50-58. doi: 10.1016/j.ijcard.2016.04.163. Epub 2016 May 13. |
| Effects of metformin on survival outcomes of lung cancer patients with type 2 diabetes mellitus: a meta-analysis. | Tian RH, Zhang YG, Wu Z, Liu X, Yang JW, Ji HL. | Clin Transl Oncol. 2016 Jun;18(6):641-9. doi: 10.1007/s12094-015-1412-x. Epub 2015 Oct 12. |
| Effects of Sitagliptin on Lipid Profiles in Patients With Type 2 Diabetes Mellitus: A Meta-analysis of Randomized Clinical Trials. | Fan M, Li Y, Zhang S. | Medicine (Baltimore). 2016 Jan;95(2):e2386. doi: 10.1097/MD.0000000000002386. Review. |
| Effects of sodium-glucose cotransporter-2 inhibitors on cardiovascular events, death, and major safety outcomes in adults with type 2 diabetes: a systematic review and meta-analysis. | Wu JH, Foote C, Blomster J, Toyama T, Perkovic V, SundstrÃ¶m J, Neal B. | Lancet Diabetes Endocrinol. 2016 May;4(5):411-9. doi: 10.1016/S2213-8587(16)00052-8. Epub 2016 Mar 18. Review. Erratum in: Lancet Diabetes Endocrinol. 2016 Sep;4(9):e9. |
| Effects of tea or tea extract on metabolic profiles in patients with type 2 diabetes mellitus: a meta-analysis of ten randomized controlled trials. | Li Y, Wang C, Huai Q, Guo F, Liu L, Feng R, Sun C. | Diabetes Metab Res Rev. 2016 Jan;32(1):2-10. doi: 10.1002/dmrr.2641. Epub 2015 Apr 21. Review. |
| Effects on All-cause Mortality and Cardiovascular Outcomes in Patients With Type 2 Diabetes by Comparing Insulin With Oral Hypoglycemic Agent Therapy: A Meta-analysis of Randomized Controlled Trials. | Li J, Tong Y, Zhang Y, Tang L, Lv Q, Zhang F, Hu R, Tong N. | Clin Ther. 2016 Feb;38(2):372-386.e6. doi: 10.1016/j.clinthera.2015.12.006. Epub 2016 Jan 7. Review. |
| Efficacy and safety of canagliflozin in patients with type 2 diabetes: A meta-analysis of randomized controlled trials. | Xiong W, Xiao MY, Zhang M, Chang F. | Medicine (Baltimore). 2016 Nov;95(48):e5473. |
| Efficacy and safety of canagliflozin in type 2 diabetes mellitus: systematic review of randomized controlled trials. | Parveen R, Agarwal NB, Kaushal N, Mali G, Raisuddin S. | Expert Opin Pharmacother. 2016;17(1):105-15. doi: 10.1517/14656566.2016.1109629. Epub 2015 Dec 9. Review. |
| Efficacy and safety of dulaglutide in patients with type 2 diabetes: a meta-analysis and systematic review. | Zhang L, Zhang M, Zhang Y, Tong N. | Sci Rep. 2016 Jan 8;6:18904. doi: 10.1038/srep18904. Review. |
| Efficacy and safety of empagliflozin as add-on to metformin for type 2 diabetes: a systematic review and meta-analysis. | Zhong X, Lai D, Ye Y, Yang X, Yu B, Huang Y. | Eur J Clin Pharmacol. 2016 Jun;72(6):655-63. doi: 10.1007/s00228-016-2010-8. Epub 2016 Feb 1. Review. |
| Efficacy and safety of insulin in type 2 diabetes: meta-analysis of randomised controlled trials. | Erpeldinger S, Rehman MB, Berkhout C, Pigache C, Zerbib Y, Regnault F, GuÃ©rin E, Supper I, Cornu C, KassaÃ¯ B, Gueyffier F, Boussageon R. | BMC Endocr Disord. 2016 Jul 8;16(1):39. doi: 10.1186/s12902-016-0120-z. |
| Efficacy and safety of insulin-GLP-1 receptor agonists combination in type 2 diabetes mellitus: a systematic review. | Cimmaruta D, Maiorino MI, Scavone C, Sportiello L, Rossi F, Giugliano D, Esposito K, Capuano A. | Expert Opin Drug Saf. 2016 Dec;15(sup2):77-83. Review. |
| Efficacy and safety of once-weekly glucagon-like peptide-1 receptor agonists compared with exenatide and liraglutide in type 2 diabetes: a systemic review of randomised controlled trials | Xue X., Ren Z., Zhang A., Yang Q., Zhang W., Liu F. | International Journal of Clinical Practice (2016) 70:8 (649-656). Date of Publication: 1 Aug 2016 |
| Efficacy and safety of saxagliptin and metformin as initial combination therapy in patients with type 2 diabetes: A meta-analysis | Tan X.-Y., Lu Y., Xuan L.-P., Huang Y.-M., Hu J.-B. | International Journal of Clinical and Experimental Medicine (2016) 9:10 (18816-18823) Article Number: IJCEM0031236. Date of Publication: 30 Oct 2016 |
| Efficacy and safety of sitagliptin in patients with type 2 diabetes mellitus: A meta-analysis | Zhao J.-Y., Wang H.-P., Wang H.-J., Yao J.-M., Wu X.-Y., Dong J.-J., Liao L. | International Journal of Clinical and Experimental Medicine (2016) 9:6 (11202-11210). Date of Publication: 30 Jun 2016 |
| Efficacy and safety of the addition of a dipeptidyl peptidase-4 inhibitor to insulin therapy in patients with type 2 diabetes: A systematic review and meta-analysis. | Kim YG, Min SH, Hahn S, Oh TJ, Park KS, Cho YM. | Diabetes Res Clin Pract. 2016 Jun;116:86-95. doi: 10.1016/j.diabres.2016.03.011. Epub 2016 Apr 23. Review. |
| Efficacy and safety of vildagliptin combined with metformin in the treatment of type 2 diabetes mellitus: A systematic review and meta-analysis | Xu T., Zhan M., Jiang X. | International Journal of Clinical and Experimental Medicine (2016) 9:6 (9495-9503). Date of Publication: 30 Jun 2016 |
| Efficacy of canagliflozin combined with antidiabetic drugs in treating type¬†2 diabetes mellitus: Meta-analysis of randomized control trials. | Meng Q, Shen Y, Liu D, Jiang F. | J Diabetes Investig. 2016 May;7(3):359-65. doi: 10.1111/jdi.12417. Epub 2015 Sep 22. |
| Efficacy, safety and impact on β-cell function of dipeptidyl peptidase-4 inhibitors plus metformin combination therapy in patients with type 2 diabetes and the difference between Asians and Caucasians: a meta-analysis | Gao W., Wang Q., Yu S. | Journal of Endocrinological Investigation (2016) 39:9 (1061-1074). Date of Publication: 1 Sep 2016 |
| Elevated serum magnesium associated with SGLT2 inhibitor use in type 2 diabetes patients: a meta-analysis of randomised controlled trials. | Tang H, Zhang X, Zhang J, Li Y, Del Gobbo LC, Zhai S, Song Y. | Diabetologia. 2016 Dec;59(12):2546-2551. Epub 2016 Sep 15. |
| Glucagon-like peptide-1 receptor agonists and heart failure in type 2 diabetes: systematic review and meta-analysis of randomized and observational studies. | Li L, Li S, Liu J, Deng K, Busse JW, Vandvik PO, Wong E, Sohani ZN, Bala MM, Rios LP, Malaga G, Ebrahim S, Shen J, Zhang L, Zhao P, Chen Q, Wang Y, Guyatt GH, Sun X. | BMC Cardiovasc Disord. 2016 May 11;16:91. doi: 10.1186/s12872-016-0260-0. Review. |
| Incretin-based therapies for the treatment of non-alcoholic fatty liver disease: A systematic review and meta-analysis. | Carbone LJ, Angus PW, Yeomans ND. | J Gastroenterol Hepatol. 2016 Jan;31(1):23-31. doi: 10.1111/jgh.13026. Review. |
| Metformin Is Associated With Slightly Reduced Risk of Colorectal Cancer and Moderate Survival Benefits in Diabetes Mellitus: A Meta-Analysis. | He XK, Su TT, Si JM, Sun LM. | Medicine (Baltimore). 2016 Feb;95(7):e2749. doi: 10.1097/MD.0000000000002749. Review. |
| Pioglitazone utilization, efficacy & safety in Indian type 2 diabetic patients: A systematic review & comparison with European Medicines Agency Assessment Report. | Pai SA, Kshirsagar NA. | Indian J Med Res. 2016 Nov;144(5):672-681. doi: 10.4103/ijmr.IJMR_650_15. Review. |
| Reduced colorectal cancer incidence in type 2 diabetic patients treated with metformin: a meta-analysis. | Nie Z, Zhu H, Gu M. | Pharm Biol. 2016 Nov;54(11):2636-2642. Epub 2016 May 9. |
| Relationship of Serum Adiponectin Levels and Metformin Therapy in Patients with Type 2 Diabetes | Su J.-R., Lu Z.-H., Su Y., Zhao N., Dong C.-L., Sun L., Zhao S.-F., Li Y. | Hormone and Metabolic Research (2016) 48:2 (92-98). Date of Publication: 25 Jan 2016 |
| Safety and efficacy of dipeptidyl peptidase-4 inhibitors vs sulfonylurea in metformin-based combination therapy for type 2 diabetes mellitus: Systematic review and meta-analysis. | Foroutan N, Muratov S, Levine M. | Clin Invest Med. 2016 Apr 2;39(2):E48-62. Review. |
| SGLT 2 inhibitors compared with sitagliptin as add-on therapy to metformin in type 2 diabetes: A systematic review and metaanalysis | Sevald C.A., Jackson J.D., McAna J.F. | Value in Health (2016) 19:3 (A197). Date of Publication: May 2016 |
| Short-term and long-term effects of dipeptidyl peptidase-4 inhibitors in type 2 diabetes mellitus patients with renal impairment: a meta-analysis of randomized controlled trials. | Li R, Wang R, Li H, Sun S, Zou M, Cheng G. | Diabetes Metab Res Rev. 2016 Sep;32(6):460-9. doi: 10.1002/dmrr.2731. Epub 2015 Nov 11. Review. |
| Spotlight on empagliflozin/metformin fixed-dose combination for the treatment of type 2 diabetes: A systematic review | Kedia R., Kulkarni S., Ross M., Shivaswamy V. | Patient Preference and Adherence (2016) 10 (1999-2006). Date of Publication: 30 Sep 2016 |
| Systematic review and meta-analysis of vildagliptin for treatment of type 2 diabetes. | Bekiari E, Rizava C, Athanasiadou E, Papatheodorou K, Liakos A, Karagiannis T, Mainou M, Rika M, Boura P, Tsapas A. | Endocrine. 2016 Jun;52(3):458-80. doi: 10.1007/s12020-015-0841-1. Epub 2015 Dec 29. Review. |
| The efficacy and safety of dipeptidyl peptidase-4 inhibitors for treatment of type 2 diabetes mellitus patients with severe renal impairment: a meta-analysis. | Chen M, Liu Y, Jin J, He Q. | Ren Fail. 2016;38(4):581-7. doi: 10.3109/0886022X.2016.1149682. Epub 2016 Feb 26. |
| The efficacy and safety of liraglutide added to metformin in patients with diabetes: a meta-analysis of randomized controlled trials. | Gu J, Meng X, Guo Y, Wang L, Zheng H, Liu Y, Wu B, Wang D. | Sci Rep. 2016 Sep 7;6:32714. doi: 10.1038/srep32714. |
| The Efficacy of Ginseng-Related Therapies in Type 2 Diabetes Mellitus: An Updated Systematic Review and Meta-analysis. | Gui QF, Xu ZR, Xu KY, Yang YM. | Medicine (Baltimore). 2016 Feb;95(6):e2584. doi: 10.1097/MD.0000000000002584. Review. |
| The Use of Exenatide in Managing Markers of Cardiovascular Risk in Patients with Type 2 Diabetes: A Systematic Review. | Ojo O. | Int J Environ Res Public Health. 2016 Sep 23;13(10). pii: E941. doi: 10.3390/ijerph13100941. Review. |
| Therapeutic Concentrations of Metformin: A Systematic Review. | Kajbaf F, De Broe ME, Lalau JD. | Clin Pharmacokinet. 2016 Apr;55(4):439-59. doi: 10.1007/s40262-015-0323-x. Review. |
| Therapeutic effect of canagliflozin on type 2 diabetes mellitus: A systematic review and meta-analysis | Zhong M., Yang L., Chen X., Shen X. | International Journal of Clinical and Experimental Medicine (2016) 9:5 (7807-7817). Date of Publication: 30 May 2016 |
| Tumour Risk with Once-Weekly Glucagon-Like Peptide-1 Receptor Agonists in Type 2 Diabetes Mellitus Patients: A Systematic Review. | Guo X, Yang Q, Dong J, Liao L, Zhang W, Liu F. | Clin Drug Investig. 2016 Jun;36(6):433-41. doi: 10.1007/s40261-016-0389-8. Review. |
| Predictors of efficacy of GLP-1 agonists and DPP-4 inhibitors: A systematic review. | Bihan H, Ng WL, Magliano DJ, Shaw JE. | Diabetes Res Clin Pract. 2016 Nov;121:27-34. doi: 10.1016/j.diabres.2016.08.011. Epub 2016 Aug 26. Review. |
| A meta-analysis comparing clinical effects of short- or long-acting GLP-1 receptor agonists versus insulin treatment from head-to-head studies in type 2 diabetic patients. | Abd El Aziz MS, Kahle M, Meier JJ, Nauck MA. | Diabetes Obes Metab. 2017 Feb;19(2):216-227. doi: 10.1111/dom.12804. Epub 2016 Nov 29. |
| A Meta-Analysis of the Efficacy of Glucagon-Like Peptide-1 Receptor Agonists on Nonalcoholic Fatty Liver Disease in Patients with Type 2 Diabetes Mellitus | Tang Z.-H., Cai L., Li J.-X., Zhao M.-Y. | Chinese Pharmaceutical Journal (2017) 52:6 (500-505). Date of Publication: 22 Mar 2017 |
| A systematic review of the benefits and harms of dipeptidyl peptidase-4 inhibitor for chronic kidney disease. | Kamiya H. | Hemodial Int. 2017 Jan;21(1):72-83. doi: 10.1111/hdi.12438. Epub 2016 Jul 19. Review. |
| Adverse drug effects observed with vildagliptin versus pioglitazone or rosiglitazone in the treatment of patients with type 2 diabetes mellitus: A systematic review and meta-analysis of randomized controlled trials | Bundhun P.K., Janoo G., Teeluck A.R., Huang F. | BMC Pharmacology and Toxicology (2017) 18:1 Article Number: 66. Date of Publication: 23 Oct 2017 |
| Adverse drug events observed in patients with type 2 diabetes mellitus treated with 100¬†mg versus 300¬†mg canagliflozin: a systematic review and meta-analysis of published randomized controlled trials. | Bundhun PK, Janoo G, Huang F. | BMC Pharmacol Toxicol. 2017 Apr 16;18(1):19. doi: 10.1186/s40360-017-0126-9. Review. |
| Association between metformin and the risk of gastric cancer in patients with type 2 diabetes mellitus: A meta-analysis of cohort studies | Zhou X.-L., Xue W.-H., Ding X.-F., Li L.-F., Dou M.-M., Zhang W.-J., Lv Z., Fan Z.-R., Zhao J., Wang L.-X. | Oncotarget (2017) 8:33 (55622-55631). Date of Publication: 2017 |
| Basal insulin treatment intensification in patients with type 2 diabetes mellitus: A comprehensive systematic review of current options. | Raccah D. | Diabetes Metab. 2017 Apr;43(2):110-124. doi: 10.1016/j.diabet.2016.11.007. Epub 2017 Feb 4. Review. |
| Cancer risk of sulfonylureas in patients with type 2 diabetes mellitus: A systematic review. | Chen Y, Du L, Li L, Ma J, Geng X, Yao X, Liu G, Sun X. | J Diabetes. 2017 May;9(5):482-494. doi: 10.1111/1753-0407.12435. Epub 2016 Sep 9. Review. |
| Cardiovascular effects of dipeptidyl peptidase-4 inhibitor in diabetic patients with and without established cardiovascular disease: a meta-analysis and systematic review. | Xu S, Zhang X, Tang L, Zhang F, Tong N. | Postgrad Med. 2017 Mar;129(2):205-215. doi: 10.1080/00325481.2017.1255537. Epub 2016 Nov 17. Review. |
| Cardiovascular Safety of Incretin-Based Therapies in Type 2 Diabetes: Systematic Review of Integrated Analyses and Randomized Controlled Trials. | Mannucci E, Monami M. | Adv Ther. 2017 Jan;34(1):1-40. doi: 10.1007/s12325-016-0432-4. Epub 2016 Nov 14. Review. |
| Circulating Nesfatin-1 Levels and Type 2 Diabetes: A Systematic Review and Meta-Analysis. | Zhai T, Li SZ, Fan XT, Tian Z, Lu XQ, Dong J. | J Diabetes Res. 2017;2017:7687098. doi: 10.1155/2017/7687098. Epub 2017 Dec 28. Review. |
| Combination therapy of metformin plus dipeptidyl peptidase-4 inhibitor versus metformin plus sulfonylurea and their association with a decreased risk of cardiovascular disease in type 2 diabetes mellitus patients. | Wang F, He Y, Zhang R, Zeng Q, Zhao X. | Medicine (Baltimore). 2017 Sep;96(36):e7638. doi: 10.1097/MD.0000000000007638. |
| Dipeptidyl peptidase-4 inhibitors and risk of arthralgia: A systematic review and meta-analysis. | Men P, He N, Song C, Zhai S. | Diabetes Metab. 2017 Dec;43(6):493-500. doi: 10.1016/j.diabet.2017.05.013. Epub 2017 Aug 1. Review. |
| Effect of dipeptidyl peptidase-4 inhibitors on circulating tumor necrosis factor-Î± concentrations: A systematic review and meta-analysis of controlled trials. | Atkin SL, Katsiki N, Banach M, Mikhailidis DP, Pirro M, Sahebkar A. | J Diabetes Complications. 2017 Sep;31(9):1458-1464. doi: 10.1016/j.jdiacomp.2017.05.016. Epub 2017 Jun 3. Review. |
| Effect of dragon fruit on glycemic control in prediabetes and type 2 diabetes: A systematic review and meta-analysis. | Poolsup N, Suksomboon N, Paw NJ. | PLoS One. 2017 Sep 8;12(9):e0184577. doi: 10.1371/journal.pone.0184577. eCollection 2017. Review. |
| Effects of dipeptidyl peptidase-4 inhibitors in type 2 diabetes patients with moderate to severe chronic kidney disease: Meta-analysis of randomized controlled trials using unadjusted data. | Yang M, Wang L, Gu L, Yuan W. | J Diabetes. 2017 Dec;9(12):1107-1117. doi: 10.1111/1753-0407.12546. Epub 2017 May 29. |
| Effects of dipeptidyl peptidase-4 inhibitors on beta-cell function and insulin resistance in type 2 diabetes: meta-analysis of randomized controlled trials. | Lyu X, Zhu X, Zhao B, Du L, Chen D, Wang C, Liu G, Ran X. | Sci Rep. 2017 Mar 21;7:44865. doi: 10.1038/srep44865. |
| Effects of Oral and Non-Insulin Injectable Antidiabetic Treatment in Hypertension: A Systematic Review. | Katsi V, Georgiopoulos G, Vogiatzi G, Oikonomou D, Megapanou M, Skoumas J, Vlachopoulos C, Nihoyannopoulos P, Tousoulis D. | Curr Pharm Des. 2017;23(25):3743-3750. doi: 10.2174/1381612823666170519144841. Review. |
| Effects of SGLT2 inhibitors on UTIs and genital infections in type 2 diabetes mellitus: a systematic review and meta-analysis. | Liu J, Li L, Li S, Jia P, Deng K, Chen W, Sun X. | Sci Rep. 2017 Jun 6;7(1):2824. doi: 10.1038/s41598-017-02733-w. Review. |
| Effects of sodium glucose co-transporter-2 inhibitors on urinary tract infections and genital infections in patients with type 2 diabetes: A systematic review and meta-analysis | Liu J., Li L., Li S., Deng K., Sun X. | Value in Health (2017) 20:5 (A163). Date of Publication: 1 May 2017 |
| Effects of sodium-glucose co-transporter 2 (SGLT2) inhibition on renal function and albuminuria in patients with type 2 diabetes: A systematic review and meta-analysis | Xu L., Li Y., Lang J., Xia P., Zhao X., Wang L., Yu Y., Chen L. | PeerJ (2017) 2017:6 (e3405). Date of Publication: 2017 |
| Efficacy and safety of dulaglutide for treating patients with type-2 diabetes mellitus: Evidence based systematic review and meta-analysis of randomized controlled trials | Hussain S., Dasari A. | Value in Health (2017) 20:5 (A165). Date of Publication: 1 May 2017 |
| Efficacy and safety of empagliflozin in type 2 diabetes mellitus: a meta-analysis of randomized controlled trials. | Devi R, Mali G, Chakraborty I, Unnikrishnan MK, Abdulsalim S. | Postgrad Med. 2017 Apr;129(3):382-392. doi: 10.1080/00325481.2017.1259544. Epub 2016 Nov 25. Review. |
| Efficacy and safety of Gegen Qinlian decoction for normalizing hyperglycemia in diabetic patients: A systematic review and meta-analysis of randomized clinical trials. | Ryuk JA, Lixia M, Cao S, Ko BS, Park S. | Complement Ther Med. 2017 Aug;33:6-13. doi: 10.1016/j.ctim.2017.05.004. Epub 2017 May 26. Review. |
| Efficacy and safety of glucagon-like peptide-1 agonists on macrovascular and microvascular events in type 2 diabetes mellitus: A meta-analysis. | Gargiulo P, Savarese G, D'Amore C, De Martino F, Lund LH, Marsico F, Dellegrottaglie S, Marciano C, Trimarco B, Perrone-Filardi P. | Nutr Metab Cardiovasc Dis. 2017 Dec;27(12):1081-1088. doi: 10.1016/j.numecd.2017.09.006. Epub 2017 Sep 28. Review. |
| Efficacy and safety of glucagon-like peptide-1 receptor agonists in type 2 diabetes: A systematic review and mixed-treatment comparison analysis. | Htike ZZ, Zaccardi F, Papamargaritis D, Webb DR, Khunti K, Davies MJ. | Diabetes Obes Metab. 2017 Apr;19(4):524-536. doi: 10.1111/dom.12849. Epub 2017 Feb 17. Review. |
| Efficacy and safety of liraglutide versus sitagliptin both in combination with metformin in patients with type 2 diabetes: A systematic review and meta-analysis. | Li M, Yang Y, Jiang D, Ying M, Wang Y, Zhao R. | Medicine (Baltimore). 2017 Sep;96(39):e8161. doi: 10.1097/MD.0000000000008161. Review. |
| Efficacy and safety of premixed insulin analogs in Asian patients with type 2 diabetes: A systematic review. | Sheu WH, Ji L, Lee WJ, Jabbar A, Han JH, Lew T. | J Diabetes Investig. 2017 Jul;8(4):518-534. doi: 10.1111/jdi.12605. Epub 2017 Mar 31. Review. |
| Efficacy of basal-bolus insulin regimens in the inpatient management of non-critically ill patients with type 2 diabetes: A systematic review and meta-analysis. | Christensen MB, Gotfredsen A, N√∏rgaard K. | Diabetes Metab Res Rev. 2017 Jul;33(5). doi: 10.1002/dmrr.2885. Epub 2017 Feb 23. Review. |
| Ethnic Difference in the Pharmacodynamics-efficacy Relationship of Dipeptidyl Peptidase-4 Inhibitors Between Japanese and non-Japanese Patients: A Systematic Review. | Ito Y, Ambe K, Kobayashi M, Tohkin M. | Clin Pharmacol Ther. 2017 Oct;102(4):701-708. doi: 10.1002/cpt.692. Epub 2017 May 27. Review. |
| Factors Related to the Glucose-Lowering Efficacy of Dipeptidyl Peptidase-4 Inhibitors: A Systematic Review and Meta-Analysis Focusing on Ethnicity and Study Regions. | Fujita K, Kaneko M, Narukawa M. | Clin Drug Investig. 2017 Mar;37(3):219-232. doi: 10.1007/s40261-016-0478-8. Review. |
| Fixed ratio combinations of glucagon like peptide 1 receptor agonists with basal insulin: a systematic review and meta-analysis. | Liakopoulou P, Liakos A, Vasilakou D, Athanasiadou E, Bekiari E, Kazakos K, Tsapas A. | Endocrine. 2017 Jun;56(3):485-494. doi: 10.1007/s12020-017-1293-6. Epub 2017 Apr 12. Review. |
| Fracture risk associated with common medications used in treating type 2 diabetes mellitus | Wolverton D., Blair M.M. | American Journal of Health-System Pharmacy (2017) 74:15 (1143-1151). Date of Publication: 1 Aug 2017 |
| Glucagon-like peptide-1 receptor agonists and atrial fibrillation: a systematic review and meta-analysis of randomised controlled trials | Monami M., Nreu B., Scatena A., Giannini S., Andreozzi F., Sesti G., Mannucci E. | Journal of Endocrinological Investigation (2017) 40:11 (1251-1258). Date of Publication: 1 Nov 2017 |
| Glucagon-like peptide-1 receptor agonists compared with basal insulins for the treatment of type 2 diabetes mellitus: a systematic review and meta-analysis. | Singh S, Wright EE Jr, Kwan AY, Thompson JC, Syed IA, Korol EE, Waser NA, Yu MB, Juneja R. | Diabetes Obes Metab. 2017 Feb;19(2):228-238. doi: 10.1111/dom.12805. Epub 2016 Dec 5. Review. |
| Impact of metformin on cardiovascular disease: a meta-analysis of randomised trials among people with type 2 diabetes. | Griffin SJ, Leaver JK, Irving GJ. | Diabetologia. 2017 Sep;60(9):1620-1629. doi: 10.1007/s00125-017-4337-9. Epub 2017 Aug 2. Review. |
| Incretin based treatments and mortality in patients with type 2 diabetes: systematic review and meta-analysis. | Liu J, Li L, Deng K, Xu C, Busse JW, Vandvik PO, Li S, Guyatt GH, Sun X. | BMJ. 2017 Jun 8;357:j2499. doi: 10.1136/bmj.j2499. Review. |
| Long-term efficacy and safety of sodium-glucose cotransporter-2 inhibitors as add-on to metformin treatment in the management of type 2 diabetes mellitus: A meta-analysis. | Li J, Gong Y, Li C, Lu Y, Liu Y, Shao Y. | Medicine (Baltimore). 2017 Jul;96(27):e7201. doi: 10.1097/MD.0000000000007201. Review. |
| Metformin and cancer in type 2 diabetes: a systematic review and comprehensive bias evaluation. | Farmer RE, Ford D, Forbes HJ, Chaturvedi N, Kaplan R, Smeeth L, Bhaskaran K. | Int J Epidemiol. 2017 Apr 1;46(2):728-744. doi: 10.1093/ije/dyw275. Review. Erratum in: Int J Epidemiol. 2017 Apr 1;46(2):745. |
| Metformin combined with acarbose vs. Single medicine in the treatment of type 2 diabetes: A meta-analysis | Liu Z., Zhao X., Sun W., Wang Y., Liu S., Kang L. | Experimental and Therapeutic Medicine (2017) 13:6 (3137-3145). Date of Publication: 1 Jun 2017 |
| Metformin Improves Overall Survival of Colorectal Cancer Patients with Diabetes: A Meta-Analysis. | Meng F, Song L, Wang W. | J Diabetes Res. 2017;2017:5063239. doi: 10.1155/2017/5063239. Epub 2017 Feb 8. Review. |
| Metformin is associated with survival benefit in pancreatic cancer patients with diabetes: a systematic review and meta-analysis. | Zhou PT, Li B, Liu FR, Zhang MC, Wang Q, Li YY, Xu C, Liu YH, Yao Y, Li D. | Oncotarget. 2017 Apr 11;8(15):25242-25250. doi: 10.18632/oncotarget.15692. Review. |
| Metformin therapy and risk of colorectal adenomas and colorectal cancer in type 2 diabetes mellitus patients: A systematic review and meta-analysis | Liu F., Yan L., Wang Z., Lu Y., Chu Y., Li X., Liu Y., Rui D., Nie S., Xiang H. | Oncotarget (2017) 8:9 (16017-16026). Date of Publication: 2017 |
| Metformin therapy and the risk of colorectal adenoma in patients with type 2 diabetes: A meta-analysis. | Hou YC, Hu Q, Huang J, Fang JY, Xiong H. | Oncotarget. 2017 Jan 31;8(5):8843-8853. doi: 10.18632/oncotarget.13633. Review. |
| Metformin Use Is Associated with Reduced Incidence and Improved Survival of Endometrial Cancer: A Meta-Analysis. | Tang YL, Zhu LY, Li Y, Yu J, Wang J, Zeng XX, Hu KX, Liu JY, Xu JX. | Biomed Res Int. 2017;2017:5905384. doi: 10.1155/2017/5905384. Epub 2017 Mar 20. Review. |
| Nigella sativa improves glucose homeostasis and serum lipids in type 2 diabetes: A systematic review and meta-analysis | Daryabeygi-Khotbehsara R., Golzarand M., Ghaffari M.P., Djafarian K. | Complementary Therapies in Medicine (2017) 35 (6-13). Date of Publication: 1 Dec 2017 |
| Once-weekly dipeptidyl peptidase-4 inhibitors for type 2 diabetes: a systematic review and meta-analysis. | Stoimenis D, Karagiannis T, Katsoula A, Athanasiadou E, Kazakos K, Bekiari E, Matthews DR, Tsapas A. | Expert Opin Pharmacother. 2017 Jun;18(9):843-851. doi: 10.1080/14656566.2017.1324848. Epub 2017 May 9. Review. |
| Pioglitazone for Secondary Stroke Prevention: A Systematic Review and Meta-Analysis. | Lee M, Saver JL, Liao HW, Lin CH, Ovbiagele B. | Stroke. 2017 Feb;48(2):388-393. doi: 10.1161/STROKEAHA.116.013977. Epub 2016 Dec 20. Review. |
| Safety and efficacy of a glucagon-like peptide-1 receptor agonist added to basal insulin therapy versus basal insulin with or without a rapid-acting insulin in patients with type 2 diabetes: results of a meta-analysis. | Wysham CH, Lin J, Kuritzky L. | Postgrad Med. 2017 May;129(4):436-445. doi: 10.1080/00325481.2017.1297669. Epub 2017 Mar 15. |
| Safety, Efficacy, and Bioavailability of Fixed-Dose Combinations in Type 2 Diabetes Mellitus: A Systematic Updated Review | Vijayakumar T.M., Jayram J., Meghana Cheekireddy V., Himaja D., Dharma Teja Y., Narayanasamy D. | Current Therapeutic Research - Clinical and Experimental (2017) 84 (4-9). Date of Publication: 2017 |
| Short- and long-term outcomes of metformin compared with insulin alone in pregnancy: a systematic review and meta-analysis. | Butalia S, Gutierrez L, Lodha A, Aitken E, Zakariasen A, Donovan L. | Diabet Med. 2017 Jan;34(1):27-36. doi: 10.1111/dme.13150. Epub 2016 Jun 8. Review. |
| Sodium-glucose co-transporter 2 inhibitors in addition to insulin therapy for management of type 2 diabetes mellitus: A meta-analysis of randomized controlled trials. | Tang H, Cui W, Li D, Wang T, Zhang J, Zhai S, Song Y. | Diabetes Obes Metab. 2017 Jan;19(1):142-147. doi: 10.1111/dom.12785. Epub 2016 Sep 29. |
| Systematic review of metformin monotherapy and dual therapy with sodium glucose co-transporter 2 inhibitor (SGLT-2) in treatment of type 2 diabetes mellitus. | Molugulu N, Yee LS, Ye YT, Khee TC, Nie LZ, Yee NJ, Yee TK, Liang TC, Kesharwani P. | Diabetes Res Clin Pract. 2017 Oct;132:157-168. doi: 10.1016/j.diabres.2017.07.025. Epub 2017 Jul 25. Review. |
| The association between metformin use and colorectal cancer survival among patients with diabetes mellitus: An updated meta-analysis | Tian S., Lei H.-B., Liu Y.-L., Chen Y., Dong W.-G. | Chronic Diseases and Translational Medicine (2017) 3:3 (169-175). Date of Publication: 1 Sep 2017 |
| Addition of dipeptidyl peptidase-4 inhibitors to insulin treatment in type 2 diabetes patients: A meta-analysis. | Yang W, Cai X, Gao X, Chen Y, Chen L, Ji L. | J Diabetes Investig. 2018 Jul;9(4):813-821. doi: 10.1111/jdi.12764. Epub 2017 Dec 5. |
| Adverse Drug Events Associated with Low-Dose (10 mg) Versus High-Dose (25 mg) Empagliflozin in Patients Treated for Type 2 Diabetes Mellitus: A Systematic Review and Meta-Analysis of Randomized Controlled Trials | Dai X., Luo Z.-C., Zhai L., Zhao W.-P., Huang F. | Diabetes Therapy (2018) 9:2 (753-770). Date of Publication: 1 Apr 2018 |
| Adverse Drug Events Associated with sitagliptin Versus canagliflozin for the Treatment of Patients with Type 2 Diabetes Mellitus: A Systematic Comparison Through a Meta-Analysis | Bundhun P.K., Huang F. | Diabetes Therapy (2018) 9:5 (1883-1895). Date of Publication: 1 Oct 2018 |
| Anti-diabetic medications and risk of macular edema in patients with type 2 diabetes: A systemic review and meta-analysis | Zhu W., Meng Y., Wu Y., Lu J. | International Journal of Clinical and Experimental Medicine (2018) 11:12 (12889-12901) Article Number: IJCEM0067558. Date of Publication: 2018 |
| Association Between Use of Sodium-Glucose Cotransporter 2 Inhibitors, Glucagon-like Peptide 1 Agonists, and Dipeptidyl Peptidase 4 Inhibitors With All-Cause Mortality in Patients With Type 2 Diabetes: A Systematic Review and Meta-analysis. | Zheng SL, Roddick AJ, Aghar-Jaffar R, Shun-Shin MJ, Francis D, Oliver N, Meeran K. | JAMA. 2018 Apr 17;319(15):1580-1591. doi: 10.1001/jama.2018.3024. Review. |
| Association of metformin intake with bladder cancer risk and oncologic outcomes in type 2 diabetes mellitus patients: A systematic review and meta-analysis. | Hu J, Chen JB, Cui Y, Zhu YW, Ren WB, Zhou X, Liu LF, Chen HQ, Zu XB. | Medicine (Baltimore). 2018 Jul;97(30):e11596. doi: 10.1097/MD.0000000000011596. Review. |
| Blood pressure lowering effects of sodium glucose transporter 2 inhibitors among adult patients with type 2 diabetes mellitus: A meta-analysis | Cahyadi A., Jimeno C.A. | Phillippine Journal of Internal Medicine (2018) 56:3 (176-188). Date of Publication: 1 Jul 2018 |
| Cardiovascular Mortality of Oral Antidiabetic Drugs Approved Before and After the 2008 US FDA Guidance for Industry: A Systemic Review and Meta-Analysis. | Goyat R, Rai P, Chang J, Ponte CD, Tan X. | Clin Drug Investig. 2018 Jun;38(6):491-501. doi: 10.1007/s40261-018-0639-z. Review. |
| Cardiovascular outcomes with glucagon-like peptide-1 receptor agonists in patients with type 2 diabetes: a meta-analysis. | Bethel MA, Patel RA, Merrill P, Lokhnygina Y, Buse JB, Mentz RJ, Pagidipati NJ, Chan JC, Gustavson SM, Iqbal N, Maggioni AP, Ã–hman P, Poulter NR, Ramachandran A, Zinman B, Hernandez AF, Holman RR; EXSCEL Study Group.. | Lancet Diabetes Endocrinol. 2018 Feb;6(2):105-113. doi: 10.1016/S2213-8587(17)30412-6. Epub 2017 Dec 6. |
| Cardiovascular safety, long-term noncardiovascular safety, and efficacy of sodium-glucose cotransporter 2 inhibitors in patients with type 2 diabetes mellitus: A systemic review and meta-analysis with trial sequential analysis | Zhang X.-L., Zhu Q.-Q., Chen Y.-H., Li X.-L., Chen F., Huang J.-A., Xu B. | Journal of the American Heart Association (2018) 7:2 Article Number: e007165. Date of Publication: 1 Jan 2018 |
| Comparing SGLT-2 inhibitors to DPP-4 inhibitors as an add-on therapy to metformin in patients with type 2 diabetes: A systematic review and meta-analysis. | Mishriky BM, Tanenberg RJ, Sewell KA, Cummings DM. | Diabetes Metab. 2018 Mar;44(2):112-120. doi: 10.1016/j.diabet.2018.01.017. Epub 2018 Feb 7. Review. |
| Comparison of dipeptidyl peptidase-4 inhibitors and pioglitazone combination therapy versus pioglitazone monotherapy in type 2 diabetes: A system review and meta-analysis. | Wang B, Sun Y, Sang Y, Liu X, Liang J. | Medicine (Baltimore). 2018 Nov;97(46):e12633. doi: 10.1097/MD.0000000000012633. Review. |
| Direct head-to-head comparison of glycaemic durability of dipeptidyl peptidase-4 inhibitors and sulphonylureas in patients with type 2 diabetes mellitus: A meta-analysis of long-term randomized controlled trials. | Chen K, Kang D, Yu M, Zhang R, Zhang Y, Chen G, Mu Y. | Diabetes Obes Metab. 2018 Apr;20(4):1029-1033. doi: 10.1111/dom.13147. Epub 2017 Dec 5. |
| Effects of Insulin Treatment with Glargine or Premixed Insulin Lispro Programs in Type 2 Diabetes Mellitus Patients: A Meta-analysis of Randomized Clinical Trials. | Sun D, Zhang X, Hou XX. | Diabetes Technol Ther. 2018 Sep;20(9):622-627. doi: 10.1089/dia.2018.0132. Epub 2018 Aug 10. |
| Effects of sodium-glucose co-transporter 2 (SGLT2) inhibitors on serum uric acid level: A meta-analysis of randomized controlled trials. | Zhao Y, Xu L, Tian D, Xia P, Zheng H, Wang L, Chen L. | Diabetes Obes Metab. 2018 Feb;20(2):458-462. doi: 10.1111/dom.13101. Epub 2017 Sep 27. |
| Effects of sodium-glucose cotransporter-2 inhibitors on cardiovascular disease, death and safety outcomes in type 2 diabetes - A systematic review. | R√•dholm K, Wu JH, Wong MG, Foote C, Fulcher G, Mahaffey KW, Perkovic V, Neal B. | Diabetes Res Clin Pract. 2018 Jun;140:118-128. doi: 10.1016/j.diabres.2018.03.027. Epub 2018 Mar 28. Review. |
| Efficacy and safety of combination therapy with SGLT2 and DPP4 inhibitors in the treatment of type 2 diabetes: A systematic review and meta-analysis. | Cho YK, Kang YM, Lee SE, Lee J, Park JY, Lee WJ, Kim YJ, Jung CH. | Diabetes Metab. 2018 Nov;44(5):393-401. doi: 10.1016/j.diabet.2018.01.011. Epub 2018 Feb 3. |
| Efficacy and safety of empagliflozin for type 2 diabetes mellitus: Meta-analysis of randomized controlled trials. | Zhang YJ, Han SL, Sun XF, Wang SX, Wang HY, Liu X, Chen L, Xia L. | Medicine (Baltimore). 2018 Oct;97(43):e12843. doi: 10.1097/MD.0000000000012843. Review. |
| Efficacy and safety of saxagliptin in patients with type 2 diabetes: A systematic review and meta-analysis. | Men P, Li XT, Tang HL, Zhai SD. | PLoS One. 2018 May 22;13(5):e0197321. doi: 10.1371/journal.pone.0197321. eCollection 2018. Review. |
| Efficacy and safety of sodium-glucose cotransporter 2 inhibitors as add-on to metformin and sulfonylurea treatment for the management of type 2 diabetes: a meta-analysis. | Li J, Shao YH, Wang XG, Gong Y, Li C, Lu Y. | Endocr J. 2018 Mar 28;65(3):335-344. doi: 10.1507/endocrj.EJ17-0372. Epub 2018 Jan 27. |
| Efficacy and safety of sodium-glucose cotransporter 2 inhibitors in patients with type 2 diabetes and moderate renal function impairment: A systematic review and meta-analysis. | Zhang L, Zhang M, Lv Q, Tong N. | Diabetes Res Clin Pract. 2018 Jun;140:295-303. doi: 10.1016/j.diabres.2018.03.047. Epub 2018 Apr 9. Review. |
| Efficacy and safety of sodium-glucose cotransporter-2 inhibitors versus dipeptidyl peptidase-4 inhibitors as monotherapy or add-on to metformin in patients with type 2 diabetes mellitus: A systematic review and meta-analysis. | Wang Z, Sun J, Han R, Fan D, Dong X, Luan Z, Xiang R, Zhao M, Yang J. | Diabetes Obes Metab. 2018 Jan;20(1):113-120. doi: 10.1111/dom.13047. Epub 2017 Aug 10. Review. |
| Efficacy of metformin on glycemic control and weight in drug-naive type 2 diabetes mellitus patients: A systematic review and meta-analysis of placebo-controlled randomized trials | Piera-Mardemootoo C., Lambert P., Faillie J.-L. | Therapie (2018). Date of Publication: 2018 |
| Empagliflozin reduces blood pressure and uric acid in patients with type 2 diabetes mellitus: a systematic review and meta-analysis | Zhao D., Liu H., Dong P. | Journal of Human Hypertension (2018). Date of Publication: 2018 |
| Ethnic Differences in Efficacy and Safety of Alogliptin: A Systematic Review and Meta-Analysis | Cai Y., Zeng T., Wen Z., Chen L. | Diabetes Therapy (2018) 9:1 (177-191). Date of Publication: 1 Feb 2018 |
| Incretin-based therapies and risk of pancreatic cancer in patients with type 2 diabetes: A meta-analysis of randomized controlled trials. | Wang H, Liu Y, Tian Q, Yang J, Lu R, Zhan S, Haukka J, Hong T. | Diabetes Obes Metab. 2018 Apr;20(4):910-920. doi: 10.1111/dom.13177. Epub 2018 Jan 3. |
| Isolated Compounds from Natural Products with Potential Antidiabetic Activity - A Systematic Review. | Munhoz ACM, Frode TS. | Curr Diabetes Rev. 2018;14(1):36-106. doi: 10.2174/1573399813666170505120621. Review. |
| Long-term risk of rosiglitazone on cardiovascular events - a systematic review and meta-analysis. | Cheng D, Gao H, Li W. | Endokrynol Pol. 2018;69(4):381-394. doi: 10.5603/EP.a2018.0036. Epub 2018 Jun 28. |
| Meta-analysis and critical review on the efficacy and safety of alpha-glucosidase inhibitors in Asian and non-Asian populations. | Gao X, Cai X, Yang W, Chen Y, Han X, Ji L. | J Diabetes Investig. 2018 Mar;9(2):321-331. doi: 10.1111/jdi.12711. Epub 2017 Aug 17. Review. |
| Meta-analysis of the association between sodium-glucose co-transporter-2 inhibitors and risk of skin cancer among patients with type 2 diabetes | Tang H., Yang K., Song Y., Han J. | Diabetes, Obesity and Metabolism (2018) 20:12 (2919-2924). Date of Publication: 1 Dec 2018 |
| Metformin use and its effect on gastric cancer in patients with type 2 diabetes: A systematic review of observational studies | Li P., Zhang C., Gao P., Chen X., Ma B., Yu D., Song Y., Wang Z. | Oncology Letters (2018) 15:1 (1191-1199). Date of Publication: 1 Jan 2018 |
| Metformin, Asian ethnicity and risk of prostate cancer in type 2 diabetes: A systematic review and meta-analysis | Chen C.B., Eskin M., Eurich D.T., Majumdar S.R., Johnson J.A. | BMC Cancer (2018) 18:1 Article Number: 65. Date of Publication: 10 Jan 2018 |
| Pioglitazone Therapy and Fractures: Systematic Review and Meta- Analysis. | Pavlova V, Filipova E, Uzunova K, Kalinov K, Vekov T. | Endocr Metab Immune Disord Drug Targets. 2018;18(5):502-507. doi: 10.2174/1871530318666180423121833. |
| PRISMA-efficacy and safety of lixisenatide for type 2 diabetes mellitus: A meta-analysis of randomized controlled trials. | Wei ZG, Wang MC, Zhang HH, Wang ZY, Wang GN, Wei FX, Zhang YW, Xu XD, Zhang YC. | Medicine (Baltimore). 2018 Dec;97(51):e13710. doi: 10.1097/MD.0000000000013710. |
| Risk of dipeptidyl peptidase-4 (DPP-4) inhibitors on site-specific cancer: A systematic review and meta-analysis. | Overbeek JA, Bakker M, van der Heijden AAWA, van Herk-Sukel MPP, Herings RMC, Nijpels G. | Diabetes Metab Res Rev. 2018 Jul;34(5):e3004. doi: 10.1002/dmrr.3004. Epub 2018 Apr 26. Review. |
| Risks of diabetic foot syndrome and amputation associated with sodium glucose co-transporter 2 inhibitors: A Meta-analysis of Randomized Controlled Trials. | Li D, Yang JY, Wang T, Shen S, Tang H. | Diabetes Metab. 2018 Nov;44(5):410-414. doi: 10.1016/j.diabet.2018.02.001. Epub 2018 Feb 13. |
| SGLT2 inhibitor plus DPP-4 inhibitor as combination therapy for type 2 diabetes: A systematic review and meta-analysis. | Li D, Shi W, Wang T, Tang H. | Diabetes Obes Metab. 2018 Aug;20(8):1972-1976. doi: 10.1111/dom.13294. Epub 2018 Apr 14. |
| SGLT-2 Inhibitors and DPP-4 Inhibitors as Second-Line Drugs in Patients with Type 2 Diabetes: A Meta-Analysis of Randomized Clinical Trials. | Wang K, Zhang Y, Zhao C, Jiang M. | Horm Metab Res. 2018 Oct;50(10):768-777. doi: 10.1055/a-0733-7919. Epub 2018 Sep 27. |
| SGLT2 inhibitors and renal outcomes in type 2 diabetes with or without renal impairment: A systematic review and meta-analysis. | Seidu S, Kunutsor SK, Cos X, Gillani S, Khunti K; For and on behalf of Primary Care Diabetes Europe.. | Prim Care Diabetes. 2018 Jun;12(3):265-283. doi: 10.1016/j.pcd.2018.02.001. Epub 2018 Feb 24. |
| SGLT2 inhibitors and risk of stroke in patients with type 2 diabetes: A systematic review and meta-analysis. | Guo M, Ding J, Li J, Wang J, Zhang T, Liu C, Huang W, Long Y, Gao C, Xu Y. | Diabetes Obes Metab. 2018 Aug;20(8):1977-1982. doi: 10.1111/dom.13295. Epub 2018 Apr 16. |
| SGLT-2 inhibitors and the risk of infections: a systematic review and meta-analysis of randomized controlled trials. | Puckrin R, Saltiel MP, Reynier P, Azoulay L, Yu OHY, Filion KB. | Acta Diabetol. 2018 May;55(5):503-514. doi: 10.1007/s00592-018-1116-0. Epub 2018 Feb 27. Review. |
| Sodium-glucose co-transporter 2 inhibitors and cardiovascular outcomes: A systematic review and meta-analysis | Usman M.S., Siddiqi T.J., Memon M.M., Khan M.S., Rawasia W.F., Talha Ayub M., Sreenivasan J., Golzar Y. | European Journal of Preventive Cardiology (2018) 25:5 (495-502). Date of Publication: 1 Mar 2018 |
| Systematic review of efficacy and safety of newer antidiabetic drugs approved from 2013 to 2017 in controlling HbA1c in diabetes patients | Palanisamy S., Yien E.L.H., Shi L.W., Si L.Y., Qi S.H., Ling L.S.C., Lun T.W., Chen Y.N. | Pharmacy (2018) 6:3 Article Number: 57. Date of Publication: 1 Sep 2018 |
| The association between insulin therapy and depression in patients with type 2 diabetes mellitus: A meta-analysis | Bai X., Liu Z., Li Z., Yan D. | BMJ Open (2018) 8:11 Article Number: e020062. Date of Publication: 1 Nov 2018 |
| The Effects of Novel Antidiabetic Drugs on Albuminuria in Type 2 DiabetesÂ Mellitus: A Systematic Review and Meta-analysis of Randomized Controlled Trials. | Luo Y, Lu K, Liu G, Wang J, Laurent I, Zhou X. | Clin Drug Investig. 2018 Dec;38(12):1089-1108. doi: 10.1007/s40261-018-0707-4. |
| Thiazolidinediones versus metformin on improving abnormal liver enzymes in patients with type 2 diabetes mellitus: A meta-analysis | Xu C., Zhao J., Zhou X., Zhang R., Xie T., Zou Z., Liao L., Dong J. | Oncotarget (2018) 9:15 (12389-12399). Date of Publication: 2018 |
| SGLT2 inhibitors for primary and secondary prevention of cardiovascular and renal outcomes in type 2 diabetes: a systematic review and meta-analysis of cardiovascular outcome trials. | Zelniker TA, Wiviott SD, Raz I, Im K, Goodrich EL, Bonaca MP, Mosenzon O, Kato ET, Cahn A, Furtado RHM, Bhatt DL, Leiter LA, McGuire DK, Wilding JPH, Sabatine MS. | Lancet. 2019 Jan 5;393(10166):31-39. doi: 10.1016/S0140-6736(18)32590-X. Epub 2018 Nov 10. |
| Combination therapy with an SGLT2 inhibitor as initial treatment for type 2 diabetes: A systematic review and meta-analysis | Milder T.Y., Stocker S.L., Shaheed C.A., McGrath-Cadell L., Samocha-Bonet D., Greenfield J.R., Day R.O. | Journal of Clinical Medicine (2019) 8:1 Article Number: 45. Date of Publication: 1 Jan 2019 |
| Effect of SGLT2 inhibitor on renal function in patients with type 2 diabetes mellitus: a systematic review and meta-analysis of randomized controlled trials | Feng C., Wu M., Chen Z., Yu X., Nie Z., Zhao Y., Bao B. | International Urology and Nephrology (2019). Date of Publication: 2019 |
| Effect of SGLT2 inhibitors on cardiovascular, renal and safety outcomes in patients with type 2 diabetes mellitus and chronic kidney disease: A systematic review and meta-analysis | Toyama T., Neuen B.L., Jun M., Ohkuma T., Neal B., Jardine M.J., Heerspink H.L., Wong M.G., Ninomiya T., Wada T., Perkovic V. | Diabetes, Obesity and Metabolism (2019). Date of Publication: 2019 |
| Inhibition of the sodium-glucose co-transporter 2 in the elderly: clinical and mechanistic insights into safety and efficacy. | Cintra R, Moura FA, Carvalho LSF, Barreto J, Tambascia M, Pecoits-Filho R, Sposito AC. | Rev Assoc Med Bras (1992). 2019 Jan;65(1):70-86. doi: 10.1590/1806-9282.65.1.70. Review. |
| Intensive treatment of hyperglycemia in the acute phase of myocardial infarction: the tenuous balance between effectiveness and safety - a systematic review and meta-analysis of randomized clinical trials. | Negreiros PH, Bau A, Nadruz W, Coelho Filho OR, Matos-Souza JR, Coelho OR, Sposito AC, Carvalho LSF. | Rev Assoc Med Bras (1992). 2019 Jan;65(1):24-32. doi: 10.1590/1806-9282.65.1.24. |
| Risk of bone fracture associated with sodium–glucose cotransporter-2 inhibitor treatment: A meta-analysis of randomized controlled trials | Cheng L., Li Y.-Y., Hu W., Bai F., Hao H.-R., Yu W.-N., Mao X.-M. | Diabetes and Metabolism (2019). Date of Publication: 2019 |
