# Supplementary material for: Reporting and methodological quality of systematic reviews and meta-analysis with protocols in Diabetes Mellitus Type II: A systematic review
Source: PLoS One. 2020 Dec 16;15(12):e0243091. doi: 10.1371/journal.pone.0243091 (PMC7743973; doi:10.1371/journal.pone.0243091)
Supplement: S1 Diagrams — (DOCX) [file pone.0243091.s003.docx]

**Figure S1. Scatterplot of PRISMA-P score with PRISMA score.**


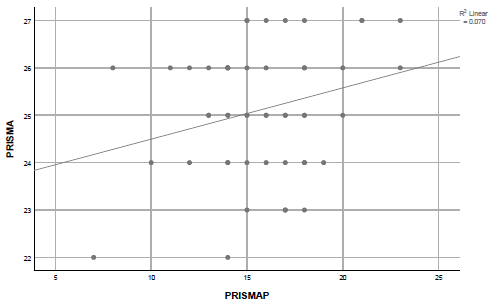


PRISMA maximum score is 27, PRISMA-P maximum score is 24

Linear regression statistical results: r=0.264; r^2^=0.070; p=0.06

**Figure S2. Scatterplot of PRISMA-P score with AMSTAR2 quality summary.**


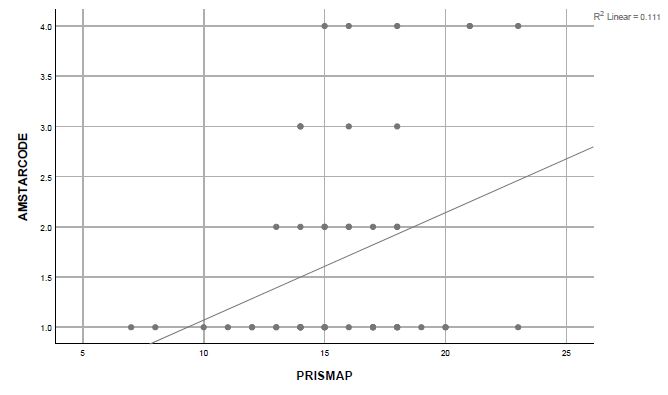


AMSTAR2 codes are as follows: 1, critically low; 2, low quality; 3, moderate quality; 4, high quality. PRISMA-P minimum score is 0, maximum score is 24

Linear regression statistical results: r=0.333; r^2^=0.11; p=0.02

**Figure S3. Scatterplot of PRISMA score with AMSTAR2 quality summary.**


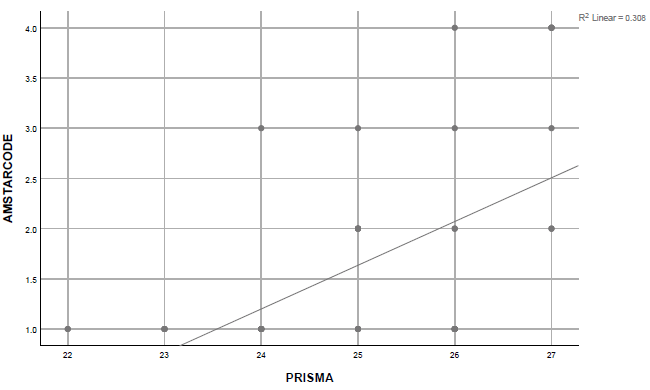


AMSTAR2 codes are as follows: 1, critically low; 2, low quality; 3, moderate quality; 4, high quality. PRISMA minimum score is 0, maximum score is 27

Linear regression statistical results: r=0.555; r^2^=0.31; p<0.01

**Figure S4. Scatterplot of PRISMA-P, PRISMA and AMSTAR2 quality summary.**

**
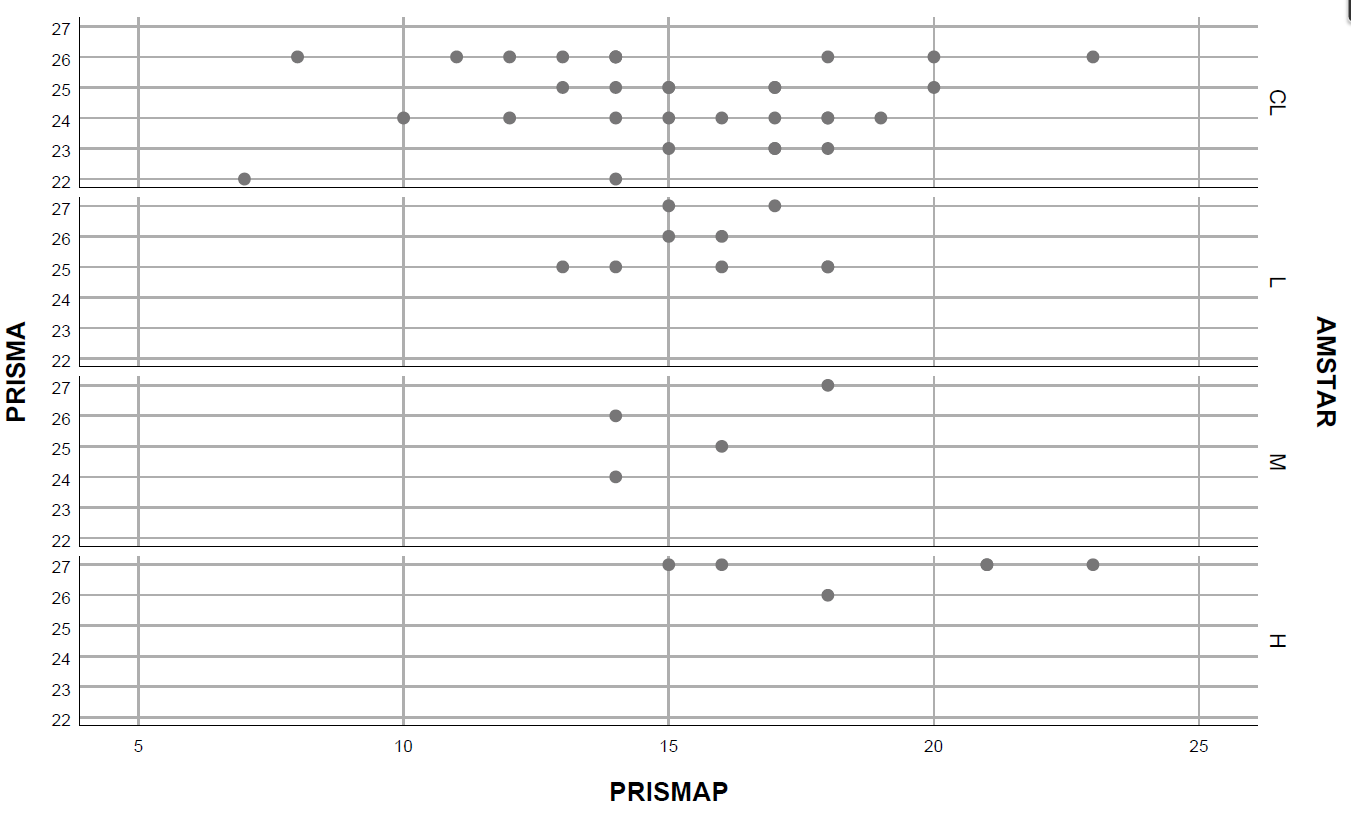
**

**Figure S5. Histogram of residuals for the multiple linear regression of PRISMA-P and PRISMA with AMSTAR2 quality summary.**
