# Supplementary material for: Reporting and methodological quality of systematic reviews and meta-analysis with protocols in Diabetes Mellitus Type II: A systematic review
Source: PLoS One. 2020 Dec 16;15(12):e0243091. doi: 10.1371/journal.pone.0243091 (PMC7743973; doi:10.1371/journal.pone.0243091)
Supplement: Attachment — Submitted filename: Type 2 Diabetes Review of SRs.docx [file pone.0243091.s005.docx]

In this review, the authors assessed the quality of published SRs in the domain of type 2 diabetes treatments. They did this by assessing fulfillment of the PRISMA, PRISMA-P and AMSTAR2 checklists. Given the importance and prevalence of diabetes as well as the emerging treatment options for this condition, and the fact that SRs are generally placed at the top of the evidence hierarchy, an evaluation of the quality of published reviews is an excellent addition to the existing body of literature, especially given the relative recency of the AMSTAR2 checklist. Overall, the manuscript is quite well-written and the authors seem to have done a thorough job with the review; nevertheless, there are a few recommendations I’ve attached below that I believe would further improve the quality of what is an already impressive manuscript.

- **General Advice:**
  - Periods should be placed after -not before- citations.
  - Although generally well-written, the manuscript would probably benefit from a revision of the grammar throughout. I have pointed out some examples of linguistic errors below, but there are a few others I haven’t included below.

**Major Revisions:**

- Intro
  - Page 5, line 77: Linguistic error “The **secondary outcomes** for this study **was** to”
- Methods:
  - Page 6, line 92 and 95: PubMed is **not a database**, it is a **search engine** designed to browse **MEDLINE**, which itself is the database.
  - Page 6, line 96 to 100: The **search strategy** does not seem very comprehensive (only two terms for diabetes were used), which may be a concerning limitation as it may limit the representativeness of the retrieved SRs. This is addressed in the limitations section of the discussion, and I commend the authors for acknowledging this point.
  - Eligibility Criteria:
    - Page 7, line 113-114: “meta-analysis” should be changed to “analys**es”** (Plural)
  - Study Selection:
    - Page 7, line 117. Linguistic error: “**By** independently **by**”
    - I believe clarifying two points in the **screening process** would benefit the manuscript.
      - First, were all records identified screened by all 3 reviewers (IE triple screening?)
      - Second, how were conflicts settled upon? (Discussion? Referral to an independent individual? (Obviously not a fourth author since the manuscript only has three authors)
  - Data Management & Extraction:
    - Page 7, line 131: Linguistic error: “Checklist **to the 1-2 articles** at a time”
    - Page 8, line 145: Linguistic error: “items which were not relevant to the study being assessed **were given we gave** the authors a full mark”
    - Page 8, line 147-148: Linguistic error: “Given full mark” to “Given **a** full mark”
  - Outcomes:
    - Line 154-155: Linguistic error: “Correlation between….compared” to “Correlation between….**and”**
    - Line 157: Linguistic error: “After **it was it was**”
    - Line 159-160: Change to “Hypothesized that **regardless of the absence or presence”** (Rather than “Despite the absence or presence”)
  - Data Synthesis:
    - Line 170: I believe it would benefit the clarity of the manuscript if the authors were to clarify what variables were entered into the multiple linear regression model (Presumably they entered PRISMA and PRISMA-P as the independent variables, but this should be made sufficiently clear to the readers).
  - Results
    - Search Results
      - Page 10, Line 191: Change “7” to “seven”.
      - The stated number of included studies is 52; however, the PRISMA diagram shows 51 included studies. It is probably a slight error, but it should be corrected both for transparency’s sake and as it affects the calculation of proportions/percentages (See later)
      - The percentage of included studies given is 14.6% (which corresponds to 51 in the PRISMA diagram, but does not correspond to 52 (14.9%) as written in the Results section)
    - Reporting and Quality Results
      - Page 11, line 198: “Items **in** which” to “Items which”
      - Line 214 to 217: I believe the authors may have made a slight mistake when calculating the **percentages**. The stated total number of included articles is 52. The number of articles of critically low quality is 32. The stated percentage is 63%, while the actual percentage (32/52) is 61.5%. I believe the same error is present in the sensitivity analysis. This may be due to the **aforementioned error in stating the number of included studies.**
    - Correlations:
      - Page 17, line 230-231: I believe the phrase “Can be used to independently predict” should be removed, as it implies a stronger correlation than there really is.
      - Line 233: I believe the manuscript would benefit if the authors clarified what variables were entered into the **multiple linear regression model.** Was it a bivariate model where PRISMA and PRISMA-P were the independent variables? If so, **the authors should clarify that the correlation score between PRISMA and PRISMA-P suggested a lack of collinearity** and therefore justified entering both variables into the same model, as most readers would (wrongly, as it turns out) assume a high degree of collinearity between these two scores.
      - I believe it the reliability of the manuscript would be improved if the authors clarified how the **linearity assumption** was verified (e.g. By providing a scatter plot), as well as, **in the case of the multiple linear model, the assumptions of homoscedasticity and the normal distribution of the residuals.**
    - Discrepancies in PRISMA and PRISMA-P Reporting
      - Table 3: “Only 1” to “Only one”
      - Table 3: Numbers add up to 51 (As stated in the PRISMA diagram), not 52 (As stated in the Methods section)
      - Table 3: I believe the quality of the manuscript would be improved by including the %s next to the raw numbers, as that would facilitate the reading and interpretation of the table.
  - Discussion
    - Line 250-251: “Lack of correlation between PRISMA and PRISMA-P”
      - I believe the **authors should not emphasize the “lack of correlation”**, as a P-value of greater than 0.05 does not definitively prove the lack of a correlation, merely the absence of sufficient evidence for said correlation. Instead, the authors should emphasize a tone of statistical insignificance rather than a definitive lack. They should also emphasize the weakness of the correlation (even if it were statistically significant) to better deliver home the point.
      - I believe exploring potential reasons for the lack of correlation would improve the quality of the paper. For instance, the authors state that the dates of publication of the PRISMA and PRISMA-P may cause the lack of said correlation. Could other factors, such as a difference in points covered by either checklist, account for the lack of correlation? Another implication may be that authors simply do not invest equivalent amounts of time and/or effort into fulfilling both checklists.
    - Line 258-259: Linguistic error: “**The higher the score of PRISMA-P and PRISMA tools results** in a higher quality categorization according to the AMSTAR-2 tool”
    - Line 260: Should be changed to “The **presence** of a correlation is intuitively correct”, as **it is not intuitive that the correlation would be relatively weak** as found by the authors (But the **existence** of a statistically significant correlation would be expected).
    - Starting from line 289, the point about limiting discrepancies to the mere presence/absence of details in the protocol/manuscript rather than actually checking for differences in the details provided is a major limitation. This is because many areas, such as statistical analysis, may be significantly modified between the protocol and the original study. The mere fact that they’re present both in the manuscript and the protocol would not warrant a “No discrepancy” judgement. Nevertheless, I commend the authors for acknowledging this limitation.
    - Line 320-321: Regarding the point about Kappa coefficients, it would be more accurate to state that low kappa ratings are common when **responses to a binary variable fall within a single category (e.g. 95% Yes 5% No), had the binary variable answers been more evenly distributed (e.g. 50% Yes 50% No) the kappa coefficient would have been more reasonable.** It is not simply that binary variables give a low kappa coefficient as implied in lines 320-321.
